# Selection and validation of chemotherapy beneficiaries among elderly nasopharyngeal carcinoma (NPC) patients treated with intensity-modulated radiation therapy (IMRT): a large real-world study

**DOI:** 10.1186/s13014-022-02095-2

**Published:** 2022-08-08

**Authors:** Yan-Ling Wu, Kai-Bin Yang, Ying Huang, Jing-Rong Shi, Qing-Shui He, Lei Chen, Wen-Fei Li, Xiao-Dan Huang, Li Lin, Yu-Pei Chen, Yan-Ping Mao, Ling-Long Tang, Jun Ma

**Affiliations:** 1 Department of Radiation Oncology, State Key Laboratory of Oncology in South China, Guangdong Key Laboratory of Nasopharyngeal Carcinoma Diagnosis and Therapy, Collaborative Innovation Center of Cancer Medicine, Sun Yat-Sen University Cancer Center, 651 Dongfeng Road East, Guangzhou, 510060 People’s Republic of China; 2 Department of Radiation Oncology, National Cancer Center/National Clinical Research Center for Cancer/Cancer Hospital and Shenzhen Hospital, Chinese Academy of Medical Sciences and Peking Union Medical College, Shenzhen, 518116 Guangdong People’s Republic of China; 3 Department of Data Mining and Analysis, Guangzhou Tianpeng Technology Co., Ltd, Zhujiang East Rd. #11, Guangzhou, 510627 People’s Republic of China

**Keywords:** Elderly patients, Nasopharyngeal carcinoma, Intensity-modulated radiation therapy, Chemotherapy, Epstein–Barr virus DNA, Recursive partitioning analysis

## Abstract

**Purpose:**

Using real-world evidence, this study aimed to identify elderly nasopharyngeal carcinoma (NPC) patients who would benefit from chemotherapy.

**Methods and materials:**

1714 elderly NPC patients between April 2007 and December 2017 were identified. Recursive partitioning analysis (RPA) was used to generate risk-stratified outcomes. Prognostic factors were performed for individual comparisons of different risk groups to assess chemotherapy benefits.

**Results:**

The median follow-up was 59.3 (0.39–170.09) months. Epstein Barr virus (EBV) DNA and T stage were included in the RPA-generated risk stratification, categorizing patients into a good-prognosis group (EBV DNA ≤ 4000 copies/mL & T1–2), and a poor-prognosis group (EBV DNA ≤ 4000 copies/mL & T3–4 and EBV DNA > 4000 copies/mL & any T). Overall survival (OS) was significantly higher in the good-prognosis group compared with the training set (HR = 0.309, 95% CI 0.184–0.517; *P* < 0.001), and validated in the testing set (HR = 0.276, 95% CI 0.113–0.670; *P* = 0.002). In the poor-prognosis group, a significantly improved OS for chemoradiotherapy (CRT) compared with RT alone was observed (HR = 0.70, 95% CI 0.55–0.88; *P* = 0.003). Patients who received induction chemotherapy (IC) + concurrent chemoradiotherapy (CCRT) and CCRT had a significantly improved OS compared with RT alone (IC + CCRT vs. RT alone: *P* = 0.002; CCRT vs. RT alone: *P* = 0.008) but not in the IC + RT group (*P* = 0.306). The 5-year OS for CRT versus RT-alone with ACE-27 scores of 0, 1 and 2 were 76.0% versus 70.0% (*P* = 0.014), 80.5% versus 68.2% (*P* = 0.150) and 58.5% versus 62.2% (*P* = 0.490), respectively; for those aged 60–64, 65–70 and ≥ 70 years old they were 80.9% versus 75.9% (*P* = 0.068), 73.3% versus 63.4% (*P* = 0.270) and 64.8% versus 67.1% (*P* = 0.820), respectively.

**Conclusions:**

For elderly NPC patients a simple screening cutoff for chemotherapy beneficiaries might be EBV DNA < 4000 copies/ml & T3–4 and EBV DNA ≥ 4000 copies/ml & any T, but not for those > 70 years old and with an ACE-27 score > 1. IC + CCRT and CCRT were effective forms of chemotherapy.

**Supplementary Information:**

The online version contains supplementary material available at 10.1186/s13014-022-02095-2.

## Introduction

Nasopharyngeal carcinoma (NPC) is rare throughout the world, yet it is particularly prevalent in southern China, with the annual incidence nearly half of the global incidence [1, 2]. Radiotherapy (RT) is the primary curative treatment for NPC. Intensity-modulated radiotherapy (IMRT) is a relatively new RT technique that significantly improves local tumor control and reduces RT-related adverse events. It has been used in NPC patients for more than ten years [3–6]. According to the National Comprehensive Cancer Network (NCCN) guidelines, the comprehensive application of chemotherapy regimens (induction, concurrent or adjuvant) in IMRT are recommended for II-IVa stage NPC patients (accounting for > 90% of all non-metastasis NPC) [3, 7].

Clinical trials, particularly randomized controlled trials (RCTs), are crucial to clinical decision-making. Notably, the target population of NPC clinical trials covers most of the adult population but often excludes elderly NPC patients (age ≥ 60 or age ≥ 65 years) due to their declining physiological function and increased comorbidity rate, as these can affect the pharmacokinetics of many chemotherapy drugs, decrease sensitivity to radiotherapy and chemotherapy, and make additional chemotherapy strategies less predictable [8–14]. Although this exclusion criterion may help to discover survival benefits, it raises possible clinical issues as to whether elderly NPC patients may also benefit from current standard chemotherapy strategies.

A recent retrospective trial including 247 elderly NPC patients (aged ≥ 60 years old) treated with IMRT showed no significantly different locoregional relapse-free survival (LRRFS), distant metastasis-free survival (DMFS), disease-specific survival (DSS) or overall survival (OS) benefits between concurrent chemoradiotherapy (CCRT) and RT alone (all *P* > 0.05) [15]. Thus, the current standard treatment strategy has many deficiencies for elderly NPC patients and research is need to establish more suited and individualized chemoradiotherapy regimens.

Due to the ageing population, the proportion of elderly NPC patients is increasing. As such, more emphasis has been placed on improving treatment benefits [16]. More precise and effective screening and treatment measures are needed to guide individualized treatment for elderly NPC patients. Currently, the tumor-node-metastases (TNM) staging system is used to guide treatment and predict prognosis, of which, only anatomical factors are taken into consideration and it is therefore, inadequate in identifying chemotherapy beneficiaries [15].

Certain biomarkers have been used to assess patients who might benefit from chemotherapy. Plasma Epstein Barr virus (EBV) DNA is one of them which has demonstrated high performance in risk stratification and individualized NPC treatment [17]. A combination of TNM stage, pre-treatment plasma EBV DNA and other critical predictors have been applied to identify chemotherapy beneficiaries in elderly NPC patients.

Here, we developed a large-scale study to establish a recursive partitioning analysis (RPA) model with clinical risk factors for OS that places elderly NPC patients into risk groups (low–high). Other prognostic factors, such as chemotherapy regimen, age, and comorbidities were performed for individual comparisons of different risk groups to assess chemotherapy benefits. This study aimed to identify elderly NPC patients that would benefit from chemotherapy in order to provide optimal comprehensive therapy.

## Materials and methods

### Study design, data source and population

As Sun Yat-sen University Cancer Center (SYSUCC) is located in southern China, this large real-world dataset represents a specific subset of endemic cases. By virtue of the well-established big-data platform (Yi-du Cloud Technology Ltd., Beijing, China), this study utilized an NPC-specific dataset. Patients aged ≥ 60 years old who were initially diagnosed between April 2007 and December 2017 with non-distant metastasis were retrospectively reviewed. A detailed introduction of the intelligence platform has been published elsewhere [18]. Patients who received adjuvant chemotherapy (n = 238), target therapy or biotherapy (n = 164), and those with uncomplete treatment data (n = 58) were excluded [19]. A total of 1714 patients were included.

All patients underwent pre-treatment evaluation, including a complete history and physical examination, and hematologic and biochemical profiles. Fiberoptic nasopharyngoscopy, nasopharynx and neck magnetic resonance imaging (MRI), chest and abdominal computed tomography (CT), and a whole-body bone scan were performed to determine the TNM stage. 18F-fluorodeoxyglucose positron emission tomography (PET) and CT (PET–CT) was used to detect distant metastases. Plasma EBV DNA titer was quantified using a real-time quantitative polymerase chain-reaction (PCR) assay as described in prior studies [20, 21]. All patients were restaged according to the 8th Edition of the American Joint Committee on Cancer/Union for International Cancer Control (AJCC/UICC) [22]. This study received approval from SYSUCC’s institutional review committee (B2020-148–01). The requirement for informed consent was waived. To ensure the integrity of this study, the original raw data have been uploaded to the Research Data Deposit platform (http://www.researchdata.org.cn) with the approval RDD number:RDDA2022454508.

### Treatment

A stratified multi-therapeutic protocol based on the 8th Edition of the AJCC/UICC staging system was used for NPC patients. Patients with stage I received IMRT alone, and patients with stage II received IMRT, with or without concurrent chemotherapy. CCRT or induction chemotherapy (IC) plus CCRT was administered to stage III-IVA patients (for chemotherapy regimens see Additional file 1). Patients received IMRT on the basis of treatment principles for NPC established by SYSUCC (see Additional file 1). The cumulative IMRT doses delivered to the primary tumor were 66–72 Gy and to the neck area was 60–70 Gy in 28 to 33 fractions at 5 fractions per week. Palliative treatment, such as chemotherapy, intracavitary brachytherapy and salvageable surgery, were provided when possible for patients who suffered from relapse or metastatic disease during follow-up.

### Evaluation of comorbidity status

The Adult Comorbidity Evaluation 27 (ACE-27) was used to evaluate comorbidity status and severity [23]. Using patient’s medical records comorbidity status was categorized as none (score 0), mild (score 1), moderate (score 2), or severe (score 3), according to the ACE 27 assessments. In cases where two or more moderate ailments occurred in different organ systems or disease groupings, the overall comorbidity score was designated as severe. As described in the Comorbidity Coding Book, comorbidities that lacked specific information were defined as mild. An overall comorbidity ranking was designated by these scores, according to the highest ranked ailment.

### Follow-up

Follow-up was measured from the day of the first-treatment up until to the last visit or death from any cause. All patients were followed up every 3 months during the first two years, every 6 months for first three years, and then annually thereafter. Each follow-up included a complete physical and an electronic nasopharyngoscopy examination. Biochemical profiles, plasma EBV DNA levels, chest X-ray/CT, abdominal ultrasound/CT, and MRI of the nasopharynx and neck were routinely performed. Additional tests were arranged when clinically indicated. PET-CT or a whole-body bone scan, or if possible, biopsy was recommended in patients with suspected clinical recurrence or metastasis.

The main endpoint was OS, which was defined as the time from the first-treatment to death due to any-cause or the latest date that the patient was alive. The secondary endpoints were DSS (from the date of the first-treatment to tumor-cause mortality), DMFS (from the date of the first-treatment to the first distant relapse date), and LRRFS (from the date of the first-treatment to the first local/regional relapse).

### Statistical analysis

SPSS version 22.0 (SPSS Inc., Chicago, IL, USA) and R version 4.0.4 (http://www.r-project.org/) were used to perform the statistical analyses and generate the figures. Continuous variables including hemoglobin (Hb), albumin, lactate dehydrogenase (LDH), and C-reactive protein (CRP) were transformed into categorical variables based on the clinical cut-off values. The pre-treatment plasma EBV DNA level was divided at the optimal cut-off determined by the receiver-operating characteristic (ROC) curve analysis. Categorical variables were described by frequency and percentage and compared using the Pearson chi-square test, while continuous variables were described by mean and standard deviation and compared using the Kruskal–Wallis rank sum test. Survival rates were calculated and compared using the Kaplan–Meier curve and log-rank tests, respectively.

Univariate and multivariate Cox regression were adopted to estimate the effect of the variables on OS, DSS, DMFS and LRRFS. Predictors with statistical significance in the univariate analysis were entered into the multivariate analysis. Statistically significant predictors for 5-year OS in the multivariate Cox regression were included in the RPA analysis using the rpart package in R to stratify patients into risk groups with significantly different prognoses [24]. Excessive branches of RPA-generated risk stratification were removed using the prune function in the rpart package for realistic clinical application [24]. The survival of patients treated with CRT or RT alone in each risk group was compared to investigate chemotherapy benefits among patients in different risk groups. Subgroup analyses stratified by age, ACE-27, and smoking were performed to explore differences in chemotherapy benefits according to these variables in different risk groups. All tests were two-sided; a *P* < 0.05 was significant.

## Results

From April 2007 to December 2017, 1,714 eligible patients were included in this study. The median age was 64 years old (range 60–85 years), with a sex (M/F) ratio of 3.16:1.00. Non-keratinizing undifferentiated NPC (WHO type III) accounted for the majority of cases (97.8%). 38.6% of patients had pre-treatment plasma EBV DNA levels greater than 4000 copies/mL. The proportion of patients classified as stage I, II, III and IV was 4.5%, 14.1%, 46.9% and 34.5%, respectively. There were 835 patients with an ACE-27 score of 0, 741 patients with a score of 1 and 138 patients a score of 2–3. Most patients underwent IC + CCRT (26.8%) or CCRT (36.9%), 10.0% underwent IC + RT, and 26.3% were treated with RT alone. The baseline characteristics are shown in Table 1.

**Table 1:** Baseline characteristics of the whole real-world dataset of elderly NPC patients

Characteristics	No. patients, N = 1714 (%)
Gender	
Male	1302 (76.0)
Female	412 (24.0)
Age at diagnosis (years)	
60–64	878 (51.2)
65–69	545 (31.8)
≥ 70	291 (17.0)
Histological type	
WHO type I–II	38 (2.2)
WHO type III	1676 (97.8)
Family history of cancer	
No	1254 (73.2)
Yes	460 (26.8)
ACE-27 (scores)	
0	835 (48.7)
1	741 (43.2)
2	138 (8.1)
Plasma EBV DNA titer (copy/mL)	
≤ 4000	1053 (61.4)
> 4000	661 (38.6)
T category	
T1	186 (10.9)
T2	253 (14.8)
T3	854 (49.8)
T4	421 (24.6)
N category	
N0	315 (18.4)
N1	748 (43.6)
N2	434 (25.3)
N3	217 (12.7)
Stage	
I	77 (4.5)
II	242 (14.1)
III	803 (46.9)
IV	592 (34.5)
Treatment	
IC + CCRT	460 (26.8)
CCRT	632 (36.9)
IC + RT	171 (10.0)
RT alone	451 (26.3)

Stage, T category and N category were determined based on the 8th edition of American Joint Committee on Cancer/International Union Against Cancer staging system

*Plasma EBV DNA* plasma Epstein–Barr Virus DNA, *NPC* nasopharyngeal carcinoma, *WHO* World Health Organization, *CCRT* concurrent chemoradiotherapy, *IC* induction chemotherapy, *RT* radiotherapy

The median follow-up time was 59.3 months (range 0.39–170.09 months), 118 (6.9%) patients developed locoregional recurrence, 189 (11.0%) had distant metastasis and 33 (1.9%) had both. 463 (27.0%) patients died, the causes of death were tumor recurrence in 287 (16.7%) patients, complications after radiotherapy in 22 (1.3%) patients, non-NPC-related causes in 149 (8.7%) patients, and unknown cause in 5 (0.3%) patients. The non-NPC-related causes included 19 (1.1%) patients who died from other cancers, 57 (3.3%) patients from other chronic diseases, 6 (0.4%) patients from accidents, and 67 (3.9%) patients from natural death. The 5-year OS, DSS, DMFS and LRRFS for the whole cohort were 77.9%, 83.6%, 86.7% and 91.3%, respectively.

### RPA-generated risk stratification

The RT alone dataset (n = 451) was randomly divided into a training set (60%) and a testing set (40%) for the explorative construction and validation of the RPA models for risk stratification. Table 2 shows the detailed clinicopathological characteristics for the RT alone training set among elderly NPC patients. The ROC curve analysis for pre-treatment plasma EBV DNA indicated that the TNM staging for elderly NPC patients could be redefined with a cut-off value of 4000 copies/mL (Additional file 1: Fig. S1). After adjustment in the multivariate analysis, plasma EBV DNA, LDH, and T stage were verified to have significant effects on OS (*P* < 0.001, *P* = 0.001, *P* = 0.007, respectively) (Table 2). Risk stratification was performed using all validated predictors in the RPA. Automaticrpart algorithms were run to realize the modification of branches. Plasma EBV DNA and T stage remained in the final model while non-essential factors were removed.

**Table 2:** Baseline clinicopathological characteristics of the training set of RT alone for elderly NPC patients

Characteristics	Train set in RT alone (N = 310) (%)	Univariate analysis of OS		Multivariate analysis of OS	
		HR (95% CI)	*P	aHR (95% CI)	*P
Gender					
Male	227 (73.2)	Reference			
Female	83 (26.8)	0.946 (0.569, 1.571)	0.829	–	–
Age at diagnosis, years					
60–64	100 (32.3)	Reference	Reference		
65–69	97 (31.3)	1.632 (0.905, 2.946)	0.104	0.015 (0.541, 1.903)	0.963
≥ 70	113 (36.5)	1.870 (1.084, 3.226)	0.024	0.115 (0.619, 2.035)	0.704
Histological type					
WHO type I–II	8 (2.6)	Reference			
WHO type III	302 (97.4)	2.403 (0.333, 17.315)	0.384	–	–
Family history of cancer					
No	223 (71.9)	Reference			
Yes	87 (28.1)	1.5 (0.941, 2.391)	0.089	–	–
ACE-27, scores					
0	136 (43.9)	Reference			
1	122 (39.4)	0.824 (0.498, 1.363)	0.451	–	–
2–3	52 (16.8)	1.662 (0.939, 2.942)	0.081	–	–
Smoking					
No	214 (69.0)	Reference			
Yes	96 (31.0)	0.968 (0.606, 1.547)	0.892	–	–
Alcohol consumption					
No	278 (89.7)	Reference			
Yes	32 (10.3)	0.422 (0.154, 1.154)	0.093	–	–
^#^Plasma EBV DNA titer, copies/mL					
≤ 4000	239 (77.1)	Reference	Reference		
> 4000	71 (22.9)	3.694 (2.335, 5.845)	< 0.001	**1.078 (1.747, 4.944)**	** < 0.001**
^#^$Hb, g/L					
< 120.0 (110.0)	21 (6.8)	1.557 (0.714, 3.398)	0.266	–	–
≥ 120.0 (110.0)	289 (93.2)	Reference			
^#^Albumin, g/L					
< 40.0	36 (11.6)	2.381 (1.35, 4.2)	0.003	0.316 (0.751, 2.506)	0.304
≥ 40.0	274 (88.4)	Reference	Reference		
^#^LDH, U/L					
≤ 250	293 (94.5)	Reference	Reference		
> 250	17 (5.5)	2.492 (1.193, 5.205)	0.015	**1.254 (1.657, 7.406)**	**0.001**
^#^CRP, mg/L					
≤ 3.00	241 (77.7)	Reference			
> 3.00	69 (22.3)	1.119 (0.669, 1.873)	0.668	–	–
T category					
T1–2	150 (48.4)	Reference	Reference		
T3–4	160 (51.6)	2.586 (1.614, 4.143)	< 0.001	**0.724 (1.217, 3.496)**	**0.007**
N category					
N0–1	242 (78.1)	Reference	Reference		
N2–3	68 (21.9)	1.77 (1.078, 2.905)	0.024	0.047 (0.614, 1.788)	0.863

Variables with statistical differences are in bold

T and N category were determined based on the 8th edition of American Joint Committee on Cancer/International Union Against Cancer staging system. Hb, albumin, LDH, and CRP were converted into categorical variables based on clinical cut-off values

*Plasma EBV DNA* plasma Epstein–Barr virus DNA, *NPC* nasopharyngeal carcinoma, *WHO* World Health Organization, *ACE-27* Adult Comorbidity Evaluation 27, *Hb* hemoglobin, *LDH* serum lactate dehydrogenase, *CRP* C-reactive protein, *OS* overall survival, *HR* hazard ratio, *aHR* adjusted hazard ratio, *95% CI* 95% confidence interval

^#^All of these variables were measured before treatment

^$^Cut-off values of Hb are 120 and 110 g/L for male and female, respectively

*P values were calculated with univariate Cox proportional-hazards model

**Fig. 1:**
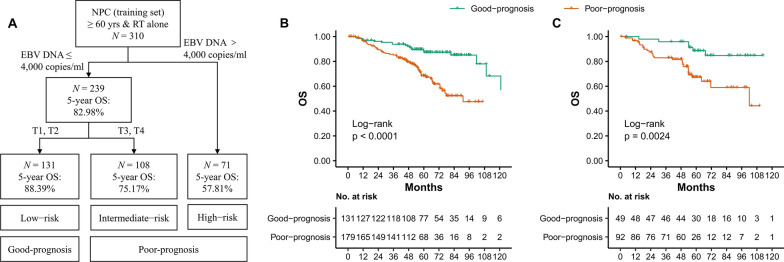
RPA-generated risk stratification for the RT alone in training set (**A**) and comparisons between the good-prognosis and poor-prognosis groups in the training set (**B**) and the testing set (**C**). RPA = recursive partitioning analysis; NPC = nasopharyngeal carcinoma; EBV = Epstein–Barr virus; HR = hazard ratio; CI = confidence interval; OS = overall survival

310 elderly NPC patients treated with RT alone were classified into low-risk (n = 131; pre-treatment plasma EBV DNA ≤ 4000 copies/mL & T1–2), intermediate-risk (n = 108; pre-treatment plasma EBV DNA ≤ 4000 copies/mL & T3–4) and high-risk groups (n = 71; pre-treatment plasma EBV DNA > 4000 copies/mL & any T) (Fig. 1A). Survival curves showed significant discrimination in OS among the groups. The corresponding 5-year OS rates were 88.39%, 75.17% and 57.81%, respectively (*P* < 0.001; Additional file 1: Fig. S2). As such, the low-risk group was considered the good-prognosis group, while the intermediate-risk and high-risk group were considered the poor-prognosis group. OS in the good-prognosis group was significantly higher than in the poor-prognosis group in the training set (HR = 0.309, 95% CI 0.184–0.517; *P* < 0.001; Fig. 1B). A similar situation was observed in the testing set (HR = 0.276, 95% CI 0.113–0.670; *P* = 0.002; Fig. 1C).

### Selection of chemotherapy and chemotherapy regimen beneficiaries

In the whole elderly NPC patient dataset, chemotherapy was administered to 73.7% (1263/1714) of the patients. In the good-prognosis group, 180 patients received radiation therapy alone and 136 patients received chemoradiotherapy (CRT). The 5-year OS, DSS, DMFS and LRRFS of the CRT group versus RT alone group were 88.1% versus 88.3% (*P* = 0.570), 91.2% versus 92.0% (*P* = 0.380), 90.9% versus 95.3% (*P* = 0.047) and 96.0% versus 94.9% (*P* = 0.580) (survival curves are shown in Additional file 1: Fig. S3).

In the poor-prognosis group, chemotherapy was administered to 80.6% (1127/1398) of the patients. The basic information is shown in Additional file 1: Table E1. Patients who received RCT in the poor-prognosis group had a significantly improved OS than those who received RT alone, (HR = 0.70, 95% CI 0.55–0.88; *P* = 0.003) and the high-risk subgroup (HR = 0.58, 95% CI 0.43–0.78; *P* < 0.001) but not in the intermediate-risk subgroup (HR = 0.80, 95% CI 0.54–1.17; *P* = 0.252). In the high-risk subgroup, CRT was validated to have significant survival benefits compared with RT alone for OS (*P* < 0.001), DSS (*P* = 0.012) and DMFS (*P* = 0.019), but not for LRRFS (*P* = 0.74) (see Additional file 1: Fig. S4).

**Fig. 2:**
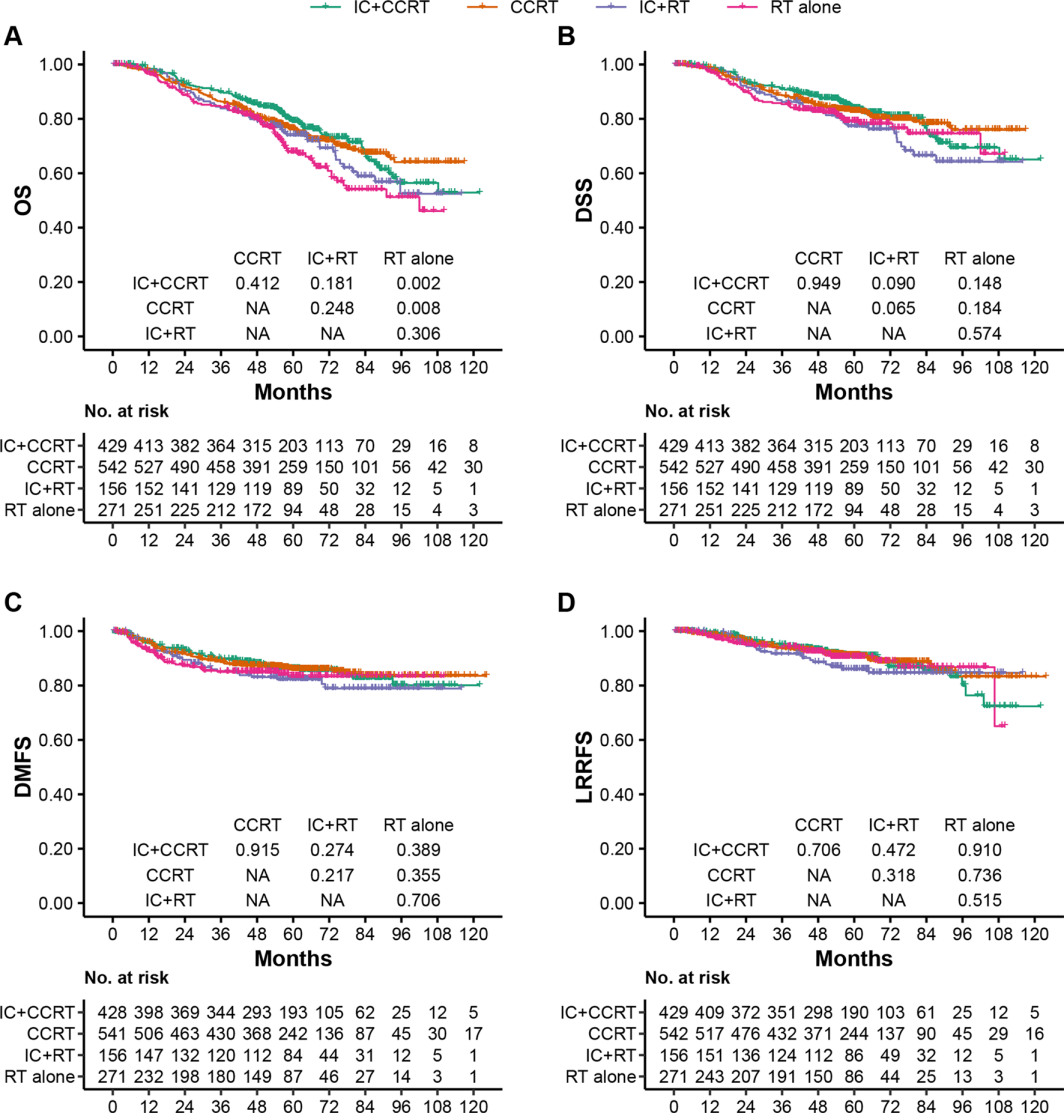
Kaplan–Meier OS (**A**), DSS, DMFS and LRRFS (**B**–**D**) curves for the poor-prognosis group between IC + CCRT, CCRT, IC + RT and RT alone. Poor-prognosis group = intermediate-risk group + high-risk group (intermediate-risk group: plasma EBV DNA titer ≤ 4000 copies/mL & T3–4; high-risk group: plasma EBV DNA titer > 4000 copies/mL & any T). EBV = Epstein–Barr virus; OS = overall survival; DSS = disease-specific survival; DMFS = distant metastasis-free survival; LRRFS = locoregional recurrence-free survival; CCRT = concurrent chemoradiotherapy; IC = induction chemotherapy; RT = radiotherapy

For chemotherapy regimens, 38.1% (429/1127) of patients received IC + CCRT, 48.1% (542/1127) received CCRT and 13.8% (156/1127) received IC + RT in the poor-prognosis group. Patients who received IC + CCRT and CCRT had significantly improved OS than those who received RT alone (IC + CCRT vs. RT alone: HR = 0.64, 95% CI 0.48–0.85; *P* = 0.002; CCRT vs. RT alone: HR = 0.70, 95% CI 0.54–0.91; *P* = 0.008) but not in the IC + RT group (IC + RT vs. RT alone: HR = 0.83, 95% CI 0.59–1.17; *P* = 0.306) (survival curves are shown in Fig. 2A). Discrimination of survival benefits were not evident compared with RT alone for DSS (IC + CCRT vs. RT alone: *P* = 0.148; CCRT vs. RT alone: *P* = 0.184; IC + RT vs. RT alone: *P* = 0.574), DMFS (IC + CCRT vs. RT alone: *P* = 0.389; CCRT vs. RT alone: *P* = 0.355; IC + RT vs. RT alone: *P* = 0.706) or LRRFS (IC + CCRT vs. RT alone: *P* = 0.910; CCRT vs. RT alone: *P* = 0.736; IC + RT vs. RT alone: *P* = 0.515) (survival curves are shown in Fig. 2B–D). More detailed information of patient chemotherapy regimens in the poor-prognosis group are listed in Additional file 1: Table E2.

### Subgroup analysis in the poor-prognosis group

To clarify whether other clinical factors impacted the prognosis of elderly NPC patients in the poor-prognosis group, univariate and multivariate analysis was performed with OS, DSS, DMFS and LRRFS (Table 3). Only the ACE-27 score was significant in predicting OS, DSS, DMFS and LRRFS (all *P* < 0.05). However, age and smoking status were significant in predicting OS (age ≥ 70 years vs. 60–64 years, *P* < 0.001; age 65–69 years vs. 60–64 years, *P* = 0.001; smoking vs. non-smoking: *P* = 0.023), as shown in Table 3.

**Table 3:** Univariate and multivariate analysis with the OS, DSS, LRRFS and DMFS for the poor-prognosis group among elderly NPC patients

Characteristic	OS		DSS		DMFS		LRRFS	
	HR (95% CI)	*P	HR (95% CI)	*P	HR (95% CI)	*P	HR (95% CI)	*P
*Univariate analysis*								
Gender								
Male	Reference		Reference		Reference		Reference	
Female	**0.677 (0.524, 0.874)**	**0.003**	0.762 (0.565, 1.028)	0.075	0.699 (0.487, 1.002)	0.052	0.750 (0.489, 1.150)	0.187
Age at diagnosis, years								
60–64	Reference		Reference		Reference		Reference	
65–69	**1.453 (1.163, 1.815)**	**0.001**	1.176 (0.899, 1.538)	0.238	1.188 (0.868, 1.625)	0.282	0.972 (0.658, 1.437)	0.887
≥ 70	**1.781 (1.385, 2.290)**	** < 0.001**	1.312 (0.953, 1.806)	0.096	1.214 (0.826, 1.786)	0.324	1.134 (0.711, 1.809)	0.597
Histological type								
WHO type I–II	Reference		Reference		Reference		Reference	
WHO type III	0.75 (0.422, 1.334)	0.328	0.659 (0.339, 1.282)	0.219	0.578 (0.272, 1.230)	0.155	1.107 (0.352, 3.481)	0.862
ACE-27(scores)								
0	Reference		Reference		Reference		Reference	
1	0.931 (0.757, 1.144)	0.494	0.868 (0.674, 1.119)	0.276	0.953 (0.705, 1.287)	0.753	**0.648 (0.447, 0.937)**	**0.021**
2	**1.885 (1.359, 2.615)**	** < 0.001**	**1.947 (1.331, 2.849)**	**0.001**	**2.117 (1.380, 3.247)**	**0.001**	1.579 (0.907, 2.749)	0.106
Family history of cancer								
No	Reference		Reference		Reference		Reference	
Yes	0.911 (0.730, 1.138)	0.412	0.87 (0.662, 1.144)	0.319	0.859 (0.621, 1.189)	0.359	1.105 (0.764, 1.598)	0.596
Smoking								
No	Reference		Reference		Reference		Reference	
Yes	**1.278 (1.053, 1.552)**	**0.013**	1.225 (0.967, 1.551)	0.092	1.319 (0.998, 1.742)	0.051	1.039 (0.740, 1.458)	0.825
Alcohol consumption								
No	Reference		Reference		Reference		Reference	
Yes	1.000 (0.770, 1.297)	0.997	0.907 (0.652, 1.261)	0.560	1.062 (0.731, 1.544)	0.751	0.811 (0.494, 1.332)	0.408
Chemotherapy								
No	Reference		Reference		Reference		Reference	
Yes	**0.697 (0.549, 0.883)**	**0.003**	0.826 (0.613, 1.114)	0.210	0.863 (0.608, 1.226)	0.411	0.975 (0.623, 1.527)	0.913
*Multivariate analysis*								
Gender								
Male	Reference		Reference		Reference		Reference	
Female	0.777 (0.587, 1.028)	0.077	0.848 (0.61, 1.18)	0.328	0.792 (0.533, 1.177)	0.249	0.754 (0.474, 1.201)	0.235
Age at diagnosis, years								
60–64	Reference		Reference		Reference		Reference	
65–69	**1.457 (1.162, 1.828)**	**0.001**	1.206 (0.917, 1.585)	0.180	1.231 (0.895, 1.694)	0.202	1.009 (0.678, 1.502)	0.965
≥ 70	**1.708 (1.302, 2.24)**	** < 0.001**	1.314 (0.931, 1.854)	0.121	1.228 (0.811, 1.86)	0.331	1.174 (0.711, 1.938)	0.53
ACE-27(scores)								
0	Reference		Reference		Reference		Reference	
1	0.944 (0.767, 1.161)	0.585	0.886 (0.687, 1.143)	0.353	0.98 (0.742, 1.323)	0.889	**0.658 (0.454, 0.954)**	**0.027**
2	**1.914 (1.349, 2.716)**	** < 0.001**	**2.112 (1.406, 3.173)**	**0.001**	**2.366 (1.5, 3.734)**	** < 0.001**	1.641 (0.911, 2.956)	0.095
Smoking								
No	Reference		Reference		Reference		Reference	
Yes	**1.306 (1.038, 1.642)**	**0.023**	1.302 (0.985, 1.722)	0.064	1.322 (0.95, 1.839)	0.098	1.018 (0.686, 1.511)	0.929
Chemotherapy								
No	Reference		Reference		Reference		Reference	
Yes	0.932 (0.71, 1.222)	0.608	1.043 (0.742, 1.465)	0.809	1.063 (0.715, 1.579)	0.764	1.177 (0.71, 1.953)	0.527

Variables with statistical differences are in bold

*NPC* nasopharyngeal carcinoma, *WHO* World Health Organization, *ACE-27* Adult Comorbidity Evaluation 27, *OS* overall survival, *DSS* disease-specific survival, *DMFS* distant metastasis-free survival, *LRRFS* locoregional recurrence-free survival, *HR* hazard ratio, *95% CI* 95% confidence interval

*P values were calculated with univariate Cox proportional-hazards model

**Fig. 3:**
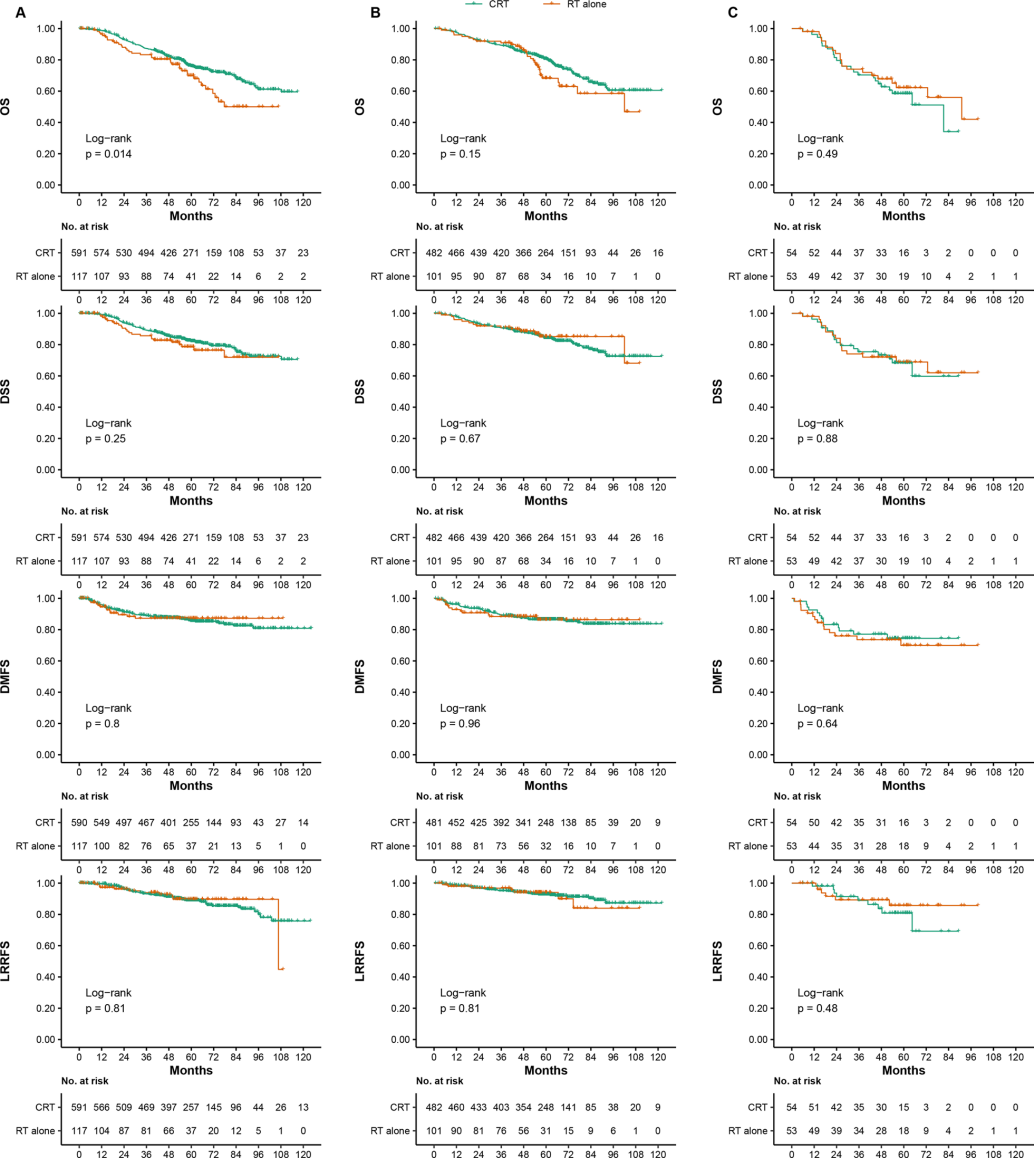
Kaplan–Meier OS, DSS, DMFS and LRRFS curves for the poor-prognosis group between CRT and RT alone with an ACE-27 score of 0 (**A**), an ACE-27 score of 1 (**B**) and ACE-27 scores of 2–3 (**C**). Poor-prognosis grou* P* = intermediate-risk group + high-risk group (intermediate-risk group: plasma EBV DNA titer ≤ 4000 copies/mL & T3–4; high-risk group: EBV DNA titer > 4000 copies/mL & any T). EBV = Epstein–Barr virus; ACE-27 = adult comorbidity evaluation 27; OS = overall survival; DSS = disease-specific survival; DMFS = distant metastasis-free survival; LRRFS = locoregional recurrence-free survival; CRT = chemoradiotherapy; RT = radiotherapy

In the poor-prognosis group, there were 591 patients with an ACE-27 score of 0, 482 patients with a score of 1 and 54 patients with a score of 2–3 in the CRT group and 117 patients with a score of 0, 101 patients with a score of 1 and 53 patients a score of 2–3 score in the RT alone group, respectively. The 5-year OS for CRT versus RT-alone with ACE-27 scores of 0, 1 and 2 were 76.0% versus 70.0% (*P* = 0.014), 80.5% versus 68.2% (*P* = 0.150) and 58.5% versus 62.2% (*P* = 0.490), respectively (survival curves are shown in Fig. 3). In the intermediate-risk group, the 5-year OS for CRT versus RT-alone with ACE-27 scores of 0, 1 and 2 were 82.9% versus 74.7% (*P* = 0.058), 86.2% versus 81.7% (*P* = 0.580) and 66.1% vs. 73.1% (*P* = 0.240), respectively (survival curves are shown in Additional file 1: Fig. S5). In the high-risk group, the 5-year OS for CRT versus RT-alone with ACE-27 scores of 0, 1 and 2 were 70.0% versus 59.6% (*P* = 0.014), 73.3% versus 54.2% (*P* = 0.037) and 48.5% versus 47.7% (*P* = 0.660), respectively (survival curves are shown in Additional file 1: Fig. S6).

**Fig. 4:**
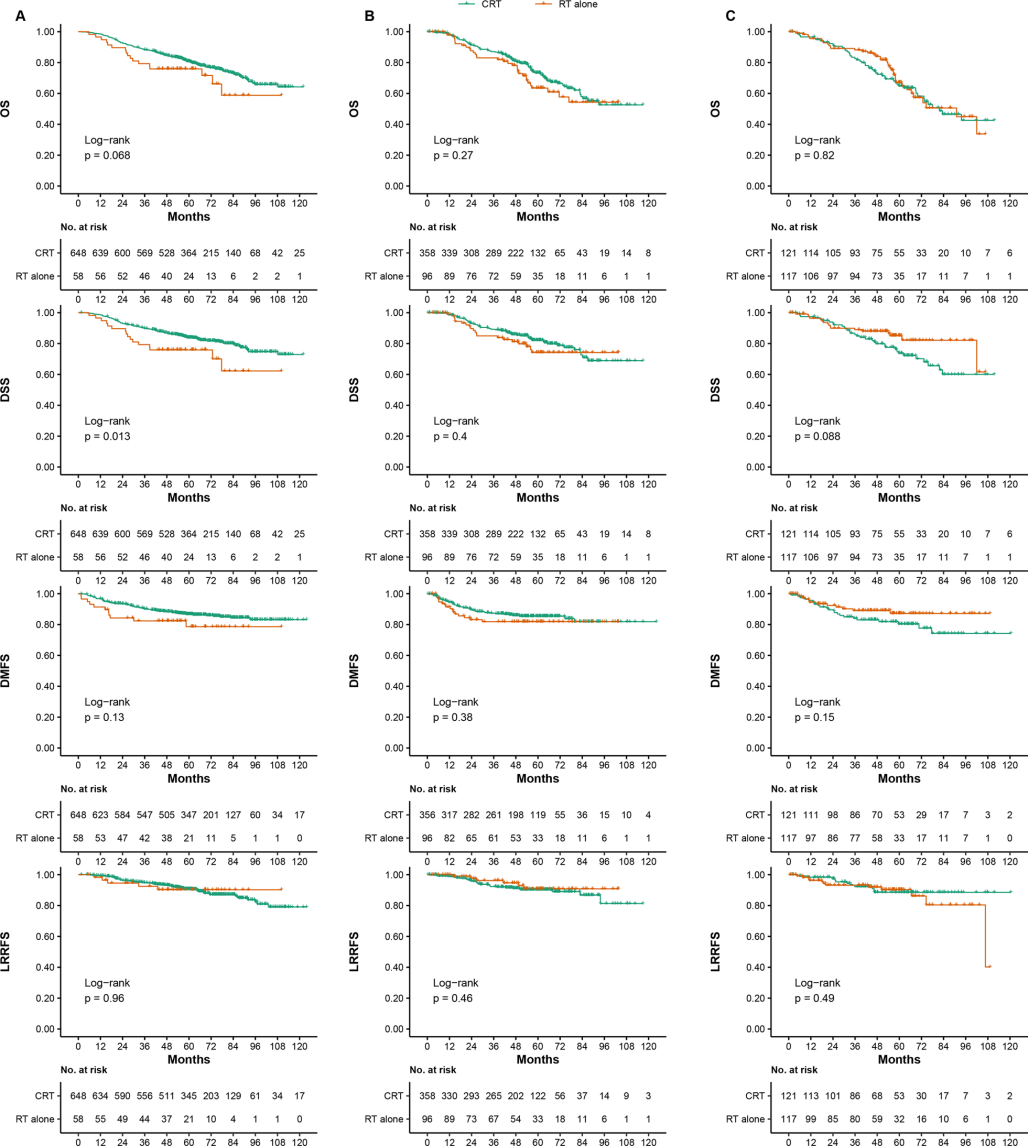
Kaplan–Meier OS, DSS, DMFS and LRRFS curves for the poor-prognosis group between CRT and RT alone for 60–64 years old (**A**), 65–70 years old (**B**) and ≥ 70 years old (**C**). Poor-prognosis grou* P* = intermediate-risk group + high-risk group (intermediate-risk group: plasma EBV DNA titer ≤ 4000 copies/mL & T3–4; high-risk group: plasma EBV DNA titer > 4000 copies/mL & any T). EBV = Epstein–Barr virus; ACE-27 = adult comorbidity evaluation 27; OS = overall survival; DSS = disease-specific survival; DMFS = distant metastasis-free survival; LRRFS = locoregional recurrence-free survival; CRT = chemoradiotherapy; RT = radiotherapy

In the poor-prognosis group, there were 648 patients aged 60–64 years old, 358 patients were 65–70 years old and 121 patients aged ≥ 70 years old in the CRT group and 58 patients aged 60–64 years old, 96 patients aged 65–70 years old and 117 patients aged ≥ 70 years old in the RT alone group, respectively. The 5-year OS for CRT versus RT-alone with 60–64, 65–70 and ≥ 70 years old were 80.9% versus 75.9% (*P* = 0.068), 73.3% versus 63.4% (*P* = 0.270) and 64.8% versus 67.1% (*P* = 0.820), respectively (survival curves are shown in Fig. 4). In the intermediate-risk group, the 5-year OS for CRT versus the RT-alone with 60–64, 65–70 and ≥ 70 years old were 85.9% versus 89.7% (*P* = 0.840), 79.7% versus 75.1% (*P* = 0.630) and 81.5% versus 70.5% (*P* = 0.088), respectively (survival curves are shown in Additional file 1: Fig. S7). In the high-risk group, the 5-year OS for CRT versus RT-alone with 60–64, 65–70 and ≥ 70 years olds were 75.9% versus 47.4% (*P* < 0.001), 66.1% versus 51.5% (*P* = 0.130) and 46.6% versus 62.2% (*P* = 0.120), respectively (survival curves are shown in Additional file 1: Fig. S8).

In the poor-prognosis group, there were 487 smokers and 640 non-smokers in the CRT group and 86 smokers and 185 non-smokers in the RT alone group, respectively. The 5-year OS for CRT versus RT-alone with smokers and non-smokers were 73.2% versus 67.0% (*P* = 0.091) and 80.1% versus 68.4% (*P* = 0.004), respectively (survival curves are shown in Additional file 1: Fig. S9). In the intermediate-risk group, the 5-year OS for CRT versus RT-alone with smoking and non-smoking were 79.1% versus 77.9% (*P* = 0.840) and 87.2% versus 76.4% (*P* = 0.099), respectively (survival curves are shown in Additional file 1: Fig. S10.). In the high-risk group, the 5-year OS for CRT versus RT-alone with smokers and non-smokers were 67.0% versus 50.5% (*P* = 0.009) and 72.8% versus 57.5% (*P* = 0.004), respectively (survival curves are shown in Supplementary Figure S11.)

## Discussion

To our knowledge, this retrospective study is the first, largest real-world dataset to use a combination of pre-treatment plasma EBV DNA and T stage to identify chemotherapy beneficiaries in elderly NPC patients. Our results revealed that patients with a plasma EBV DNA level < 4000 copies/ml & stage T3–4, and those with a plasma EBV DNA level ≥ 4000 copies/ml & any T stage are indicators for identifying elderly NPC chemotherapy beneficiaries, which is a simple and reasonable screening method. However, these indicators were not useful for those aged > 70 years old and with an ACE-27 score > 1. IC + CCRT and CCRT were effective forms of chemotherapy.

Cancer is a heterogeneous disease and the TNM staging system is the only tool to guide patient selection for chemotherapy beneficiaries in NPC. However, patients who are in the same TNM stage and undergo similar treatments, can have differing responses with more than 20% of patients having a poor effect [25–28]. These differences in prognosis might be due to biological heterogeneity, in which molecular investigations may yield biomarkers for guiding better treatment decisions for patients in different risk groups. A number of noteworthy projects have examined molecular biomarkers for NPC, such as EBV DNA, LDH, and miRNAs [29–32]. So far, EBV DNA was reported to have an independent correlation with treatment outcomes [33–37]. A previous retrospective study reported that elderly NPC patients (aged > 60 years old) receiving IMRT were not CCRT beneficiaries, which may be related to the fact that pre-treatment plasma EBV DNA levels were not included in the screening method [15]. Similarly, in this study, no differences in OS between IC + CCRT, CCRT, IC + RT and RT alone were significant in elderly NPC patients when they were not stratified (Additional file 1: Fig. S12). Two recent studies have attempted to incorporate-treatment plasma EBV DNA levels within the 8th Edition of the TNM staging system [38, 39]. Therefore, elderly NPC patients with high EBV DNA levels may be chemotherapy beneficiaries.

To select chemotherapy beneficiaries among elderly NPC patients treated by IMRT, we built a RPA model with validated predictors including plasma EBV DNA levels, TNM staging system, hematology and biochemistry (e.g. LDH), and clinical risk factors. Prognostic factors including plasma EBV DNA and T stage were predictive of treatment outcomes and categorized patients into good and poor-prognosis groups. Our results showed that chemotherapy for the poor-prognosis group was favorable to improve OS but not in the good-prognosis group (Additional file 1: Figs. S3 and S4). In our study, the most likely reason why chemotherapy benefits the poor-prognosis group patients may be the bulky or extensive tumor load, which can result in an increased risk of distant metastasis [40]. Hence, a plasma EBV DNA < 4000 copies/ml & T3–4 stage, and plasma EBV DNA ≥ 4000 copies/ml & any T stage may be able to provide a simple and reasonable screening method for chemotherapy beneficiaries in elderly NPC patients. Further prospective clinical trials are warranted.

Since IC is better tolerated, it allows for more follow-up chemotherapy, thus increasing the effectiveness in eradicating micro-metastasis [41]. The survival benefits of concurrent chemotherapy are mainly achieved by control of the locoregional tumor instead of distant metastases, due to the nature of its additive and synergistic effect with radiotherapy [42]. Our study showed that the OS benefit for the poor-prognosis group was mainly reflected in the IC + CCRT and CCRT regimens but not in the IC + RT regimens (Fig. 2). Intensive management is possible for patients receiving IC + CCRT regimens including stronger intensity treatment, longer hospital duration, better nursing care, and more supportive therapies, while intensive management itself may bring about better prognosis [43, 44]. The intermediate-risk group may prefer to avoid IC, considering IC-related expenses and toxicity, prolonged waiting times before definitive IMRT, as well as, the relatively small expected benefits (Additional file 1: Fig. S4).

Univariate and multivariate analysis with the OS, DSS, LRRFS and DMFS in the poor-prognosis group showed that ACE-27, age and smoking were significant OS predictors (Table 3). The analysis based on ACE-27 scores and age showed that the CRT group had a lower OS than the RT alone group with an ACE-27 score > 1 (58.5% vs. 62.2%) (Fig. 3) or those aged > 70 years old (64.8% vs. 67.1%) (Fig. 4). However, this result should be considered with caution, since an ACE-27 score of > 1 and the > 70 years old subgroups already had worse physiological function and lower compensative capacity. When treated with chemotherapy, the benefits might be offset by substantial effects on normal functioning with a number adverse effects [9, 10, 45]. Patients with head and neck cancer can have significant comorbidities owing to the high incidence of smoking. Patients’ baseline physical health and functioning should also be assessed when considering chemotherapy, and the related risks and benefits carefully analyzed [46].

The main strength of this study is the large-scale data from our hospital’s medical records, the actual treatment regimens and patient’s real health status. In order to be of more clinical utility, all patients in this study were restaged according the latest 8th AJCC/UICC staging system. Nevertheless, this study has several limitations. Firstly, limited by the nature of a retrospective study, there was inevitable heterogeneity in patients’ chemotherapy regimens and dosages. Secondly, as other hospitals did not provide enough patient information, external validation was not performed. Finally, the sensitivity of PCR-based EBV DNA detection is 53 to 96% accurate [47], thus the present heterogeneity is an important issue. Therefore, a large prospective multicenter clinical trial is necessary to validate our results. Nonetheless, this study provides essential information to clinicians which may inform better decision making and enable improvements in patient outcomes.

## Conclusion

Our RPA combined plasma EBV DNA and T stage, and classified elderly NPC patients into two groups with appropriate recommendations for individual therapies in real-world practice as follows: (1) plasma EBV DNA titer ≤ 4000 copies/mL & T1–2 stage: RT alone, (2) plasma EBV DNA titer ≤ 4000 copies/mL & T3–4 stage and plasma EBV DNA titer > 4000 copies/mL & any T: CRT. Patients aged > 70 years old with an ACE-27 score of > 1 should choose chemotherapy carefully. IC + CCRT and CCRT were effective forms of chemotherapy. Further validation in a larger population is still required for more specific treatment recommendations.

## Supplementary Information

**Additional file 1:** Supplementary Methods, Figures and Tables.

## Data Availability

Research data are stored in the Research Data Deposit public platform (www.researchdata.org.cn) (RDDA2022454508).

## Abbreviations

NPC: Nasopharyngeal carcinoma
IMRT: Intensity-modulated radiation therapy
RPA: Recursive partitioning analysis
EBV DNA: Epstein Barr virus DNA
CRT: Chemoradiotherapy
IC: Induction chemotherapy
CCRT: Concurrent chemoradiotherapy
RT: Radiotherapy
NCCN: National Comprehensive Cancer Network
RCTs: Randomized controlled trials
LRRFS: Locoregional relapse-free survival
DMFS: Distant metastasis-free survival
DSS: Disease-specific survival
OS: Overall survival
TNM: Tumor-node-metastases
MRI: Magnetic resonance imaging
CT: Computed tomography
PET: Positron emission tomography
PCR: Polymerase chain-reaction
AJCC/UICC: American Joint Committee on Cancer/Union for International Cancer Control
ACE-27: Adult Comorbidity Evaluation 27
Hb: Hemoglobin
LDH: Lactate dehydrogenase
CRP: C-reactive protein
ROC: Receiver-operating characteristic
