# Supplementary material for: Selection and validation of chemotherapy beneficiaries among elderly nasopharyngeal carcinoma (NPC) patients treated with intensity-modulated radiation therapy (IMRT): a large real-world study
Source: Radiat Oncol. 2022 Aug 8;17:138. doi: 10.1186/s13014-022-02095-2 (PMC9358823; doi:10.1186/s13014-022-02095-2)
Supplement: Supplementary file 1 — Additional file 1: Supplementary Methods, Figures and Tables. [file 13014_2022_2095_MOESM1_ESM.pdf]

INDEX OF SUPPLEMENTARY INFORMATION

SUMMARY……….........……...…......……...…......……..............……...………..…Page 2

SUPPLEMENTARY METHODS ..……......…...….......…..…………...……….   Page 3

SUPPLEMENTARY FIGURES ……...……………..……...….......…..….……Page 6

SUPPLEMENTARY TABLES………...……...………………..……………...….Page 18

SUMMARY

This retrospective, joint analysis based on the largest real-world dataset, is the first attempt to identify chemotherapy beneficiaries among elderly NPC patients based on plasma EBV DNA plus T stage. Our results revealed that plasma EBV DNA $<$ 4000 copies/ml & T3-4 and plasma EBV DNA ≥4000 copies/ml & any T might provide a simple and reasonable screening method for identifying elderly NPC chemotherapy beneficiaries, but not those aged >70 years old and with an ACE-27 score > 1. IC+CCRT and CCRT were effective forms of chemotherapy.

SUPPLEMENTARY METHODS

Patient restaging

All of the 1,714 patients were restaged by two experienced radiation oncologists specializing in head and neck cancers according to the 8th Edition of the American Joint Committee on Cancer/Union for International Cancer Control staging system, mainly using radiological imaging and reports as a reference. Any disagreements were resolved by consensus.

Chemotherapy regimens

Induction chemotherapy (IC) regimens consisted of TPF regimens [cisplatin (60mg/m^2) with 5-fluorouracil (600mg/m^2 over 120 h), and docetaxel (60mg/m^2)], PF regimens [cisplatin (80mg/m^2) with 5-fluorouracil (800mg/m^2/day over 120 h)], or TP regimens [cisplatin (80mg/m^2) with docetaxel (80mg/m^2)], or GP regimens [gemcitabine (1 g/m^2, d1, 8), and cisplatin (80 mg/m^2)]. All chemotherapy drugs were administered on day 1 of each 21-day cycle, except for 5-fluorouracil which was given via continuous intravenous infusion on days 1-5. Concurrent chemotherapy (CC) consisted of cisplatin/nedaplatin (80 or 100mg/m^2) given in weeks 1, 4, and 7 of radiotherapy, or cisplatin/nedaplatin (40mg/m^2) given weekly during radiotherapy.

Intensity-Modulated Radiation Therapy (IMRT)

All patients received radical high-total and fractionated-dose simultaneous modulated accelerated radiotherapy boost IMRT for 5 consecutive days every week. Target volumes were contoured using a Monaco treatment planning system, v3.02 (Elekta Medical Systems, Crawley, UK), and the dosimetry parameters estimated by radiation dose-volume histogram (DVH) calculation. The normal tissue constraints were according to RTOG 0225 and 0615 [1-2] and the Quantitative Analysis of Normal Tissue Effects in the Clinic (QUANTEC).

The prescribed doses were 66-72 Gy in 28-33 fractions to the planning target volume (PTV) of the primary gross tumor volume (GTVnx), 64-70 Gy/28-33 fractions to the PTV of the GTV of the involved lymph nodes (GTVnd), 60-63 Gy/28-33 fractions to the PTV of the high-risk clinical target volume (CTV1), and 54-56 Gy/28-33 fractions to the PTV of the low-risk clinical target volume (CTV2). CTV1 extended 5-10 mm beyond the margin of the GTVnx for potential microscopic spread, including the entire nasopharyngeal mucosa and 5 mm into the submucosal region. CTV2 extended 5-10 mm beyond the margin of the CTV1, and the potentially involved regions and lymphatic regions, unless the CTV2 was adjacent to critical organs, e.g., brain stem and spinal cord, in which case the extension distance was reduced to 3-5 mm. Radiation planning was performed utilizing high-resolution, contrast-enhanced CT under image-guided conditions with reliable contouring and a well-documented 3D dosimetry plan.

References

1. Lee N, Harris J, Garden AS, et al. Intensity-modulated radiation therapy with or without chemotherapy for nasopharyngeal carcinoma: Radiation therapy oncology group phase II trial 0225. J Clin Oncol 2009;27:3684-3690.

2. Lee NY, Zhang Q, Pfifister DG, et al. Addition of bevacizumab to standard chemoradiation for locoregionally advanced nasopharyngeal carcinoma (RTOG 0615): A phase 2 multi-institutional trial. Lancet Oncol 2012;13:172-180.

SUPPLEMENTARY FIGURES

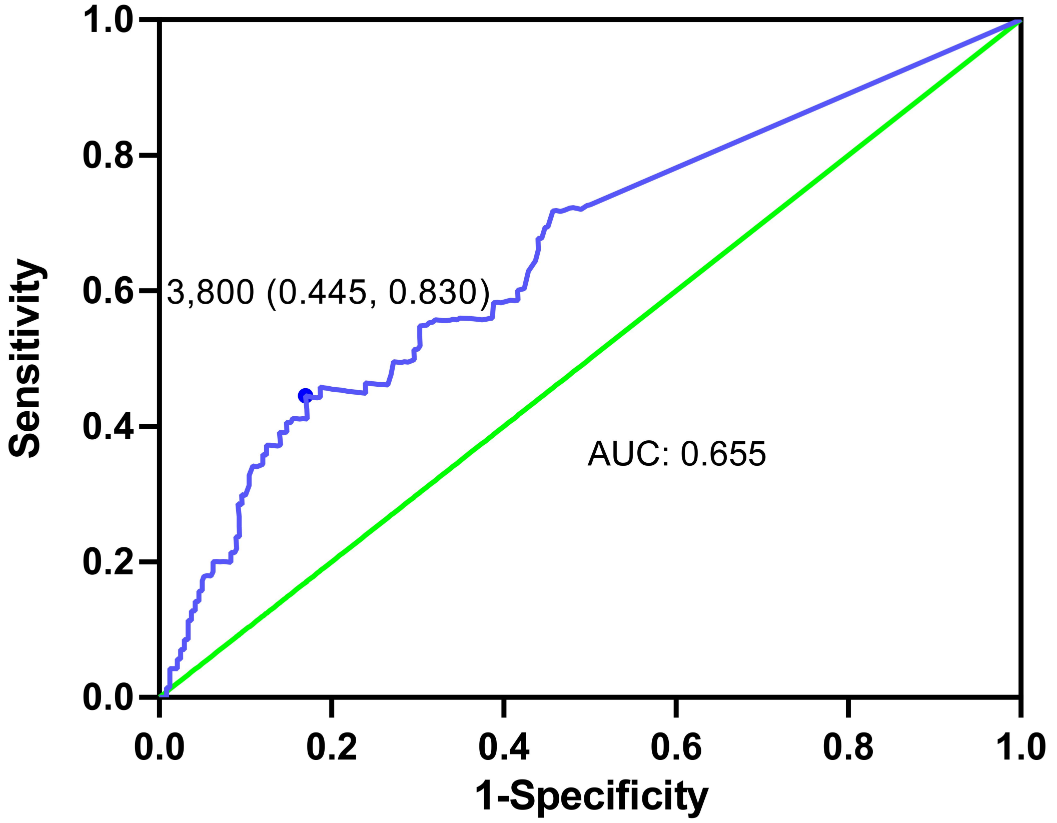

Supplementary Figure S1. The receiver operating characteristic curve analysis of pre-treatment plasma EBV DNA in the whole dataset for elderly NPC patients. The maximum value of Youden's index indicates the optimal cut-off value. The optimal cutoff was 3,800 copies/mL (sensitivity = 0.445; specificity = 0.830). NPC = nasopharyngeal carcinoma; EBV = Epstein-Barr virus; AUC = area under the curve.

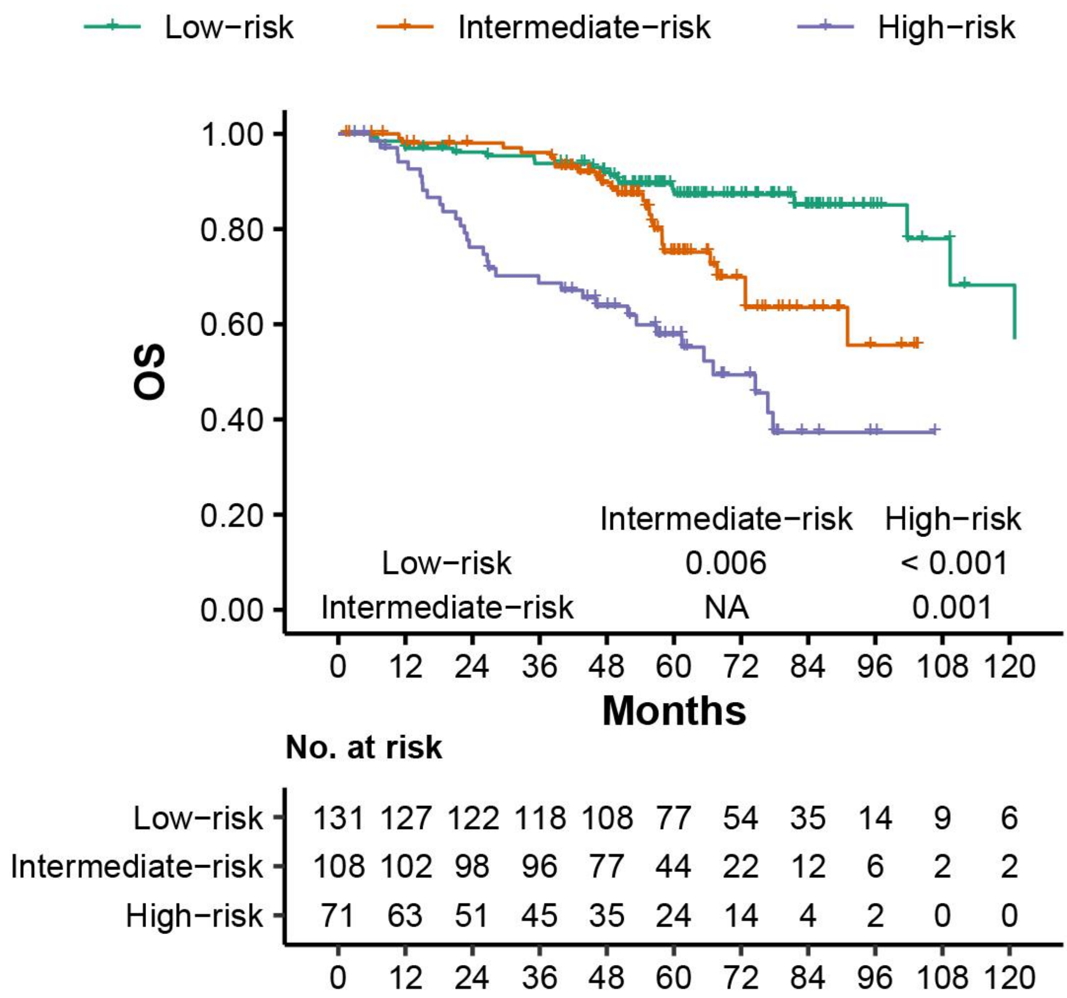

Supplementary Figure S2. Kaplan-Meier OS curves of three branches from the RPA-generated risk stratification. The three branches were the low-risk group (n = 131; plasma EBV DNA titer ≤ 4,000 copies/mL & T1-2), intermediate-risk group (n = 108; plasma EBV DNA titer ≤ 4,000 copies/mL & T3-4) and high-risk group (n = 71; plasma EBV DNA titer >4,000 copies/mL & any T). RPA = recursive partitioning analysis; OS = overall survival; EBV = Epstein-Barr virus.

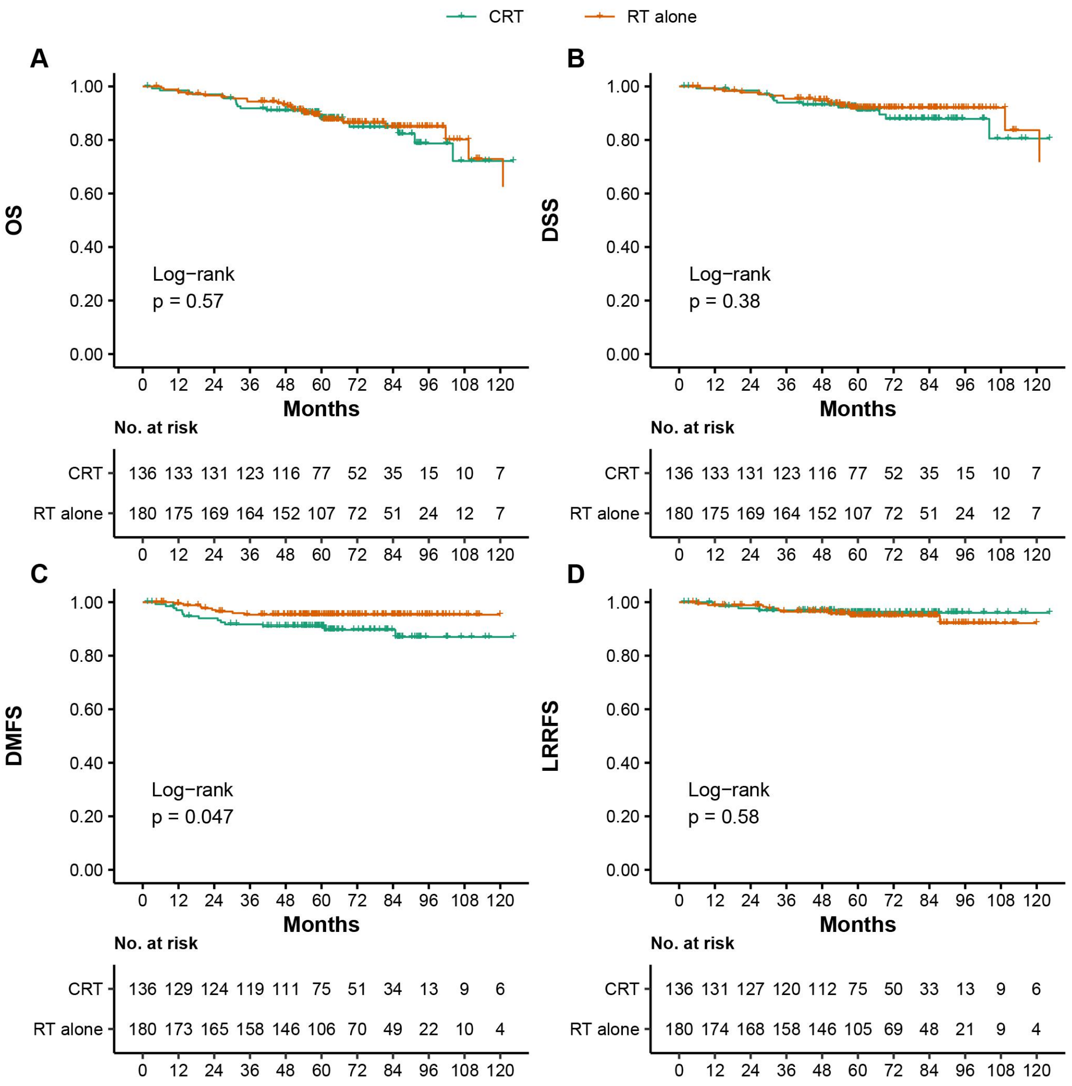

Supplementary Figure S3. Kaplan-Meier OS, DSS, DMFS and LRRFS curves for the good-prognosis group between CRT and RT alone. Good-prognosis group: plasma EBV DNA titer ≤ 4,000 copies/mL & T1-2. EBV = Epstein-Barr virus; OS = overall survival; DSS = disease-specific survival; DMFS = distant metastasis-free survival; LRRFS = locoregional recurrence-free survival; CRT = chemoradiotherapy; RT = radiotherapy.

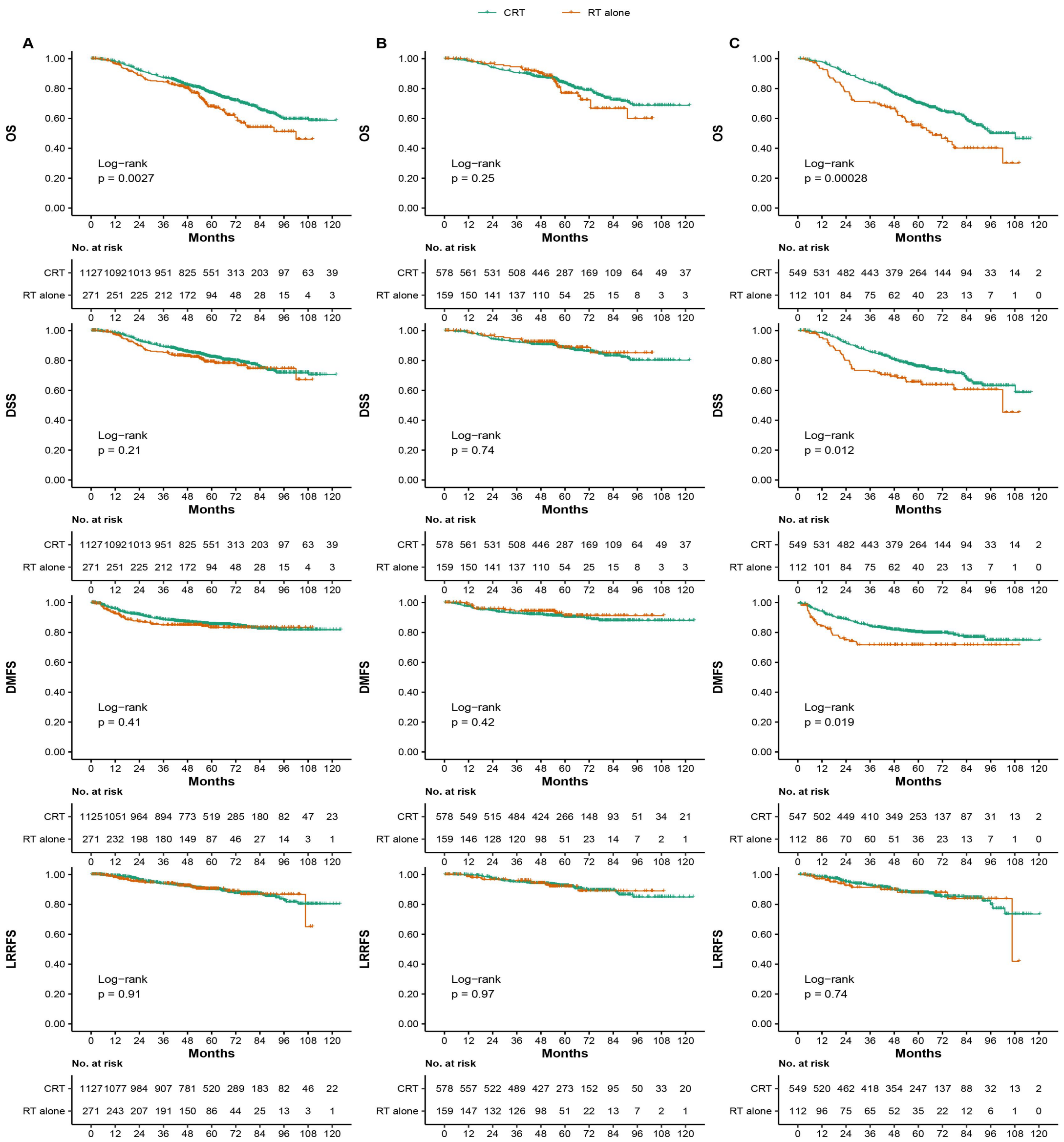

Supplementary Figure S4. Kaplan-Meier OS, DSS, DMFS and LRRFS curves between CRT and RT for the poor-prognosis group (A), intermediate-risk group (B) and high-risk group (C). Poor-prognosis group = intermediate-risk group + high-risk group (intermediate-risk group: EBV DNA titer ≤ 4,000 copies/mL& T3-4; high-risk group: EBV DNA titer > 4,000 copies/mL& any T). EBV = Epstein-Barr virus; OS = overall survival; DSS = disease-specific survival; DMFS = distant metastasis-free survival; LRRFS = locoregional recurrence-free survival; CRT = chemoradiotherapy; RT = radiotherapy.

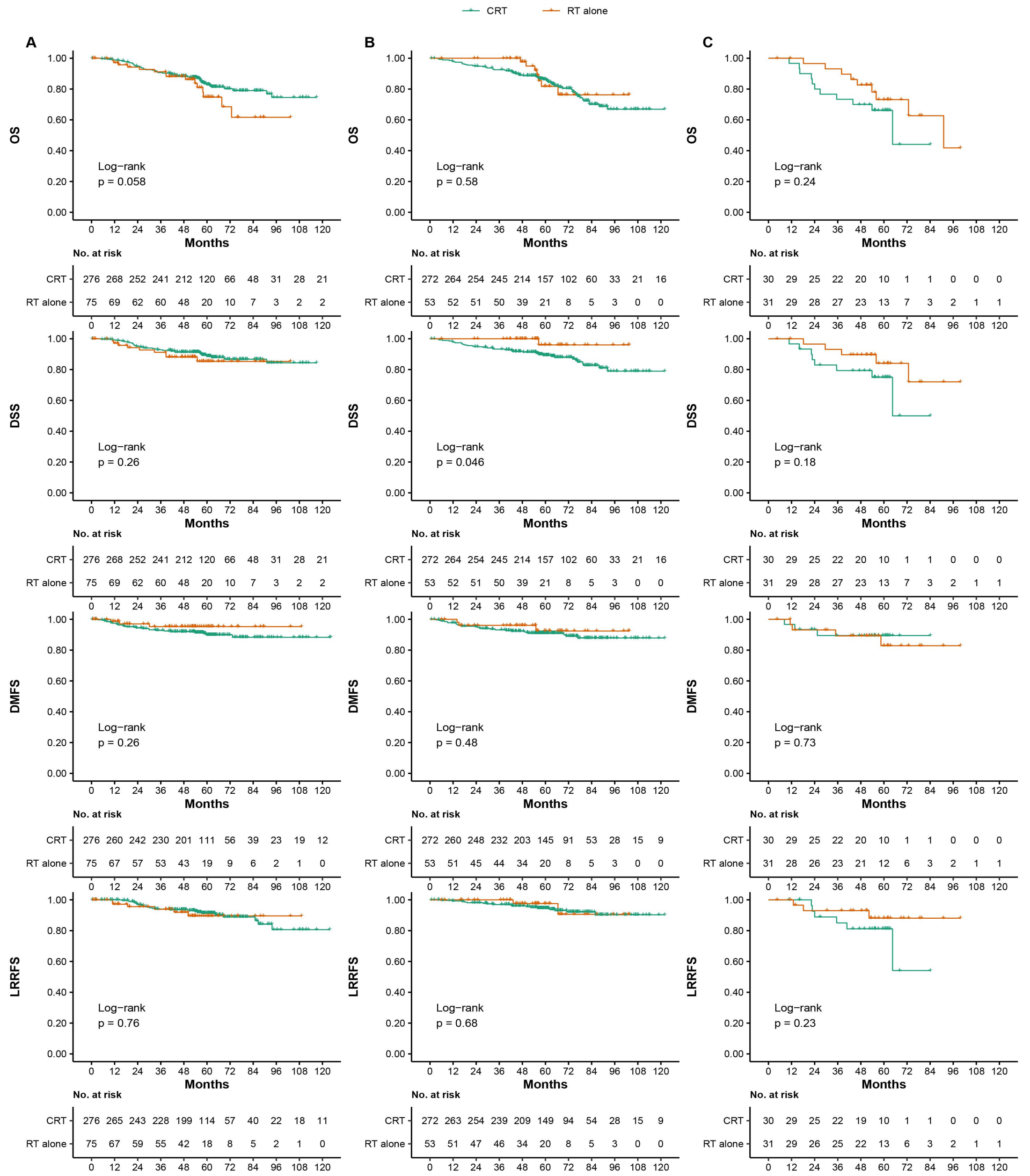

Supplementary Figure S5. Kaplan-Meier OS, DSS, DMFS and LRRFS curves in intermediate-risk group between CRT and RT alone with an ACE-27 score of 0 (A), an ACE-27 score of 1 (B) and ACE-27 scores of 2-3 (C). Intermediate-risk group: plasma EBV DNA titer ≤ 4,000 copies/mL & T3-4. EBV = Epstein-Barr virus; ACE-27 = adult comorbidity evaluation 27; OS = overall survival; DSS = disease-specific survival; DMFS = distant metastasis-free survival; LRRFS = locoregional recurrence-free survival; CRT = chemoradiotherapy; RT = radiotherapy.

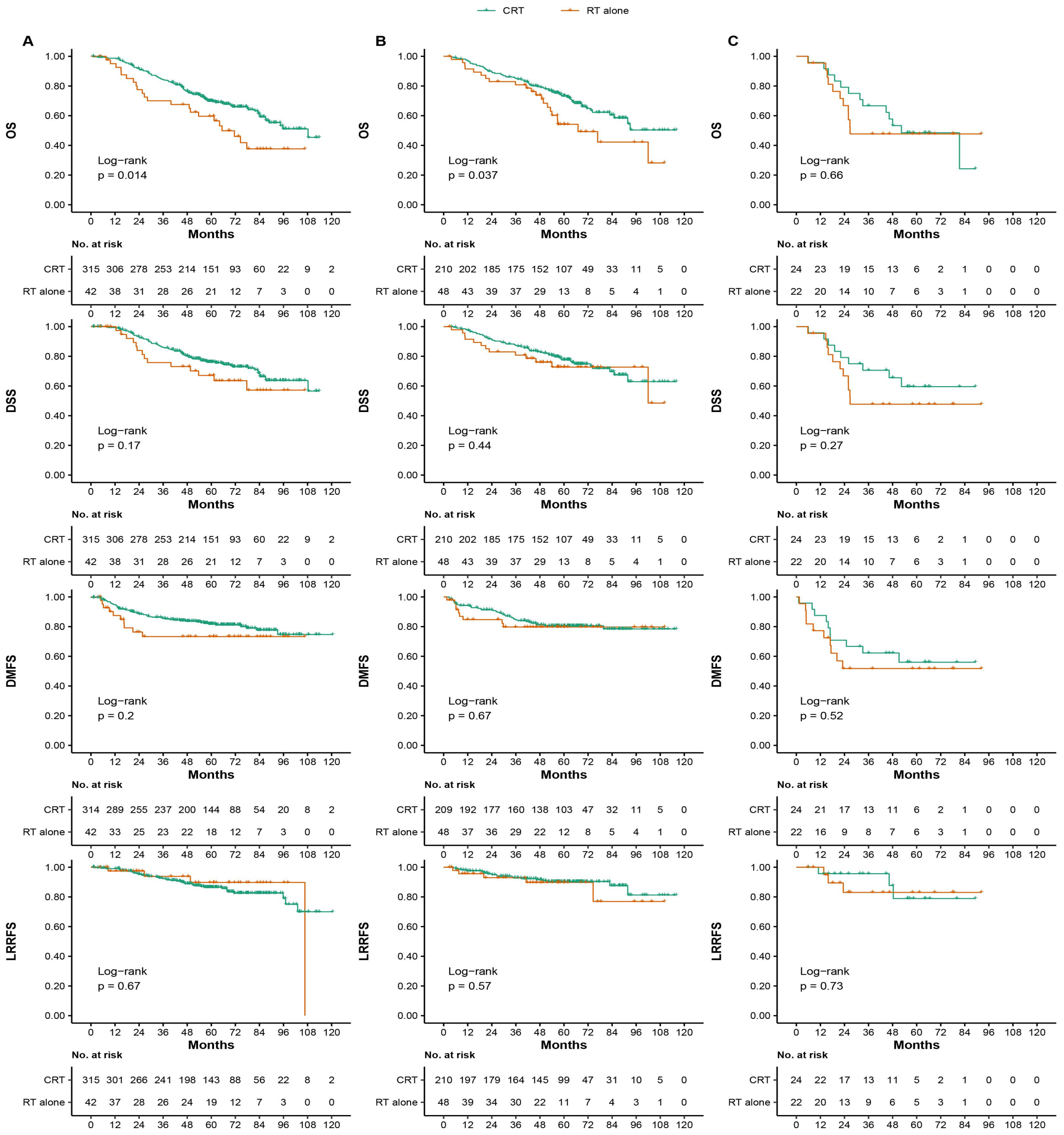

Supplementary Figure S6. Kaplan-Meier OS, DSS, DMFS and LRRFS curves for the high-risk group between CRT and RT alone with an ACE-27 score of 0 (A), an ACE-27 score of 1 score (B) and ACE-27 scores of 2-3 (C). High-risk group: plasma EBV DNA titer ≥ 4,000 copies/mL & any T. EBV = Epstein-Barr virus; ACE-27 = adult comorbidity evaluation 27; OS = overall survival; DSS = disease-specific survival; DMFS = distant metastasis-free survival; LRRFS = locoregional recurrence-free survival; CRT = chemoradiotherapy; RT = radiotherapy.

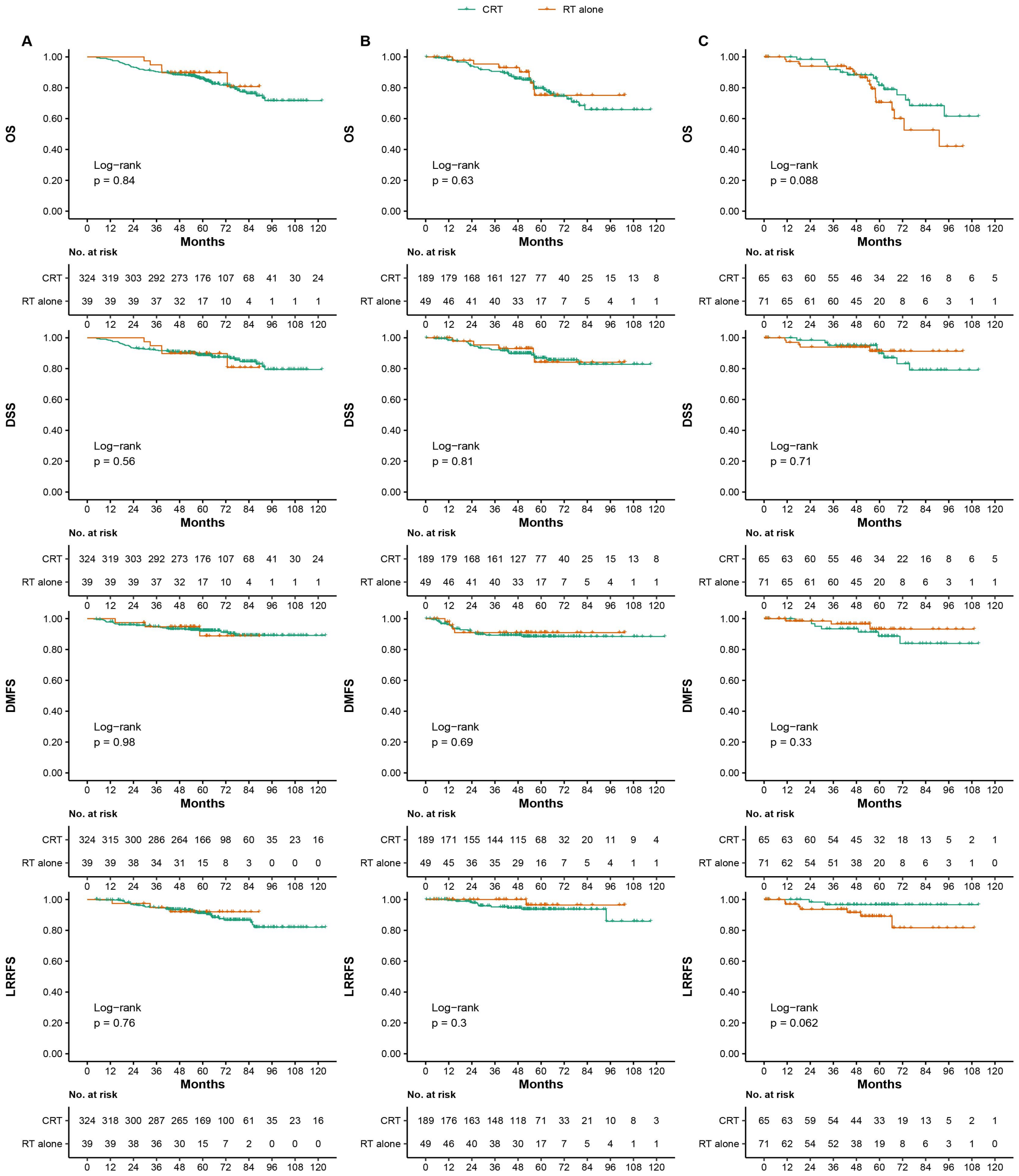

Supplementary Figure S7. Kaplan-Meier OS, DSS, DMFS and LRRFS curves for the intermediate-risk group between CRT and RT alone for aged 60-64 years old (A), 65-70 years old (B) and ≥70 years old (C). Intermediate-risk group: plasma EBV DNA titer ≤ 4,000 copies/mL & T3-4. EBV = Epstein-Barr virus; OS = overall survival; DSS = disease-specific survival; DMFS = distant metastasis-free survival; LRRFS = locoregional recurrence-free survival; CRT = chemoradiotherapy; RT = radiotherapy.

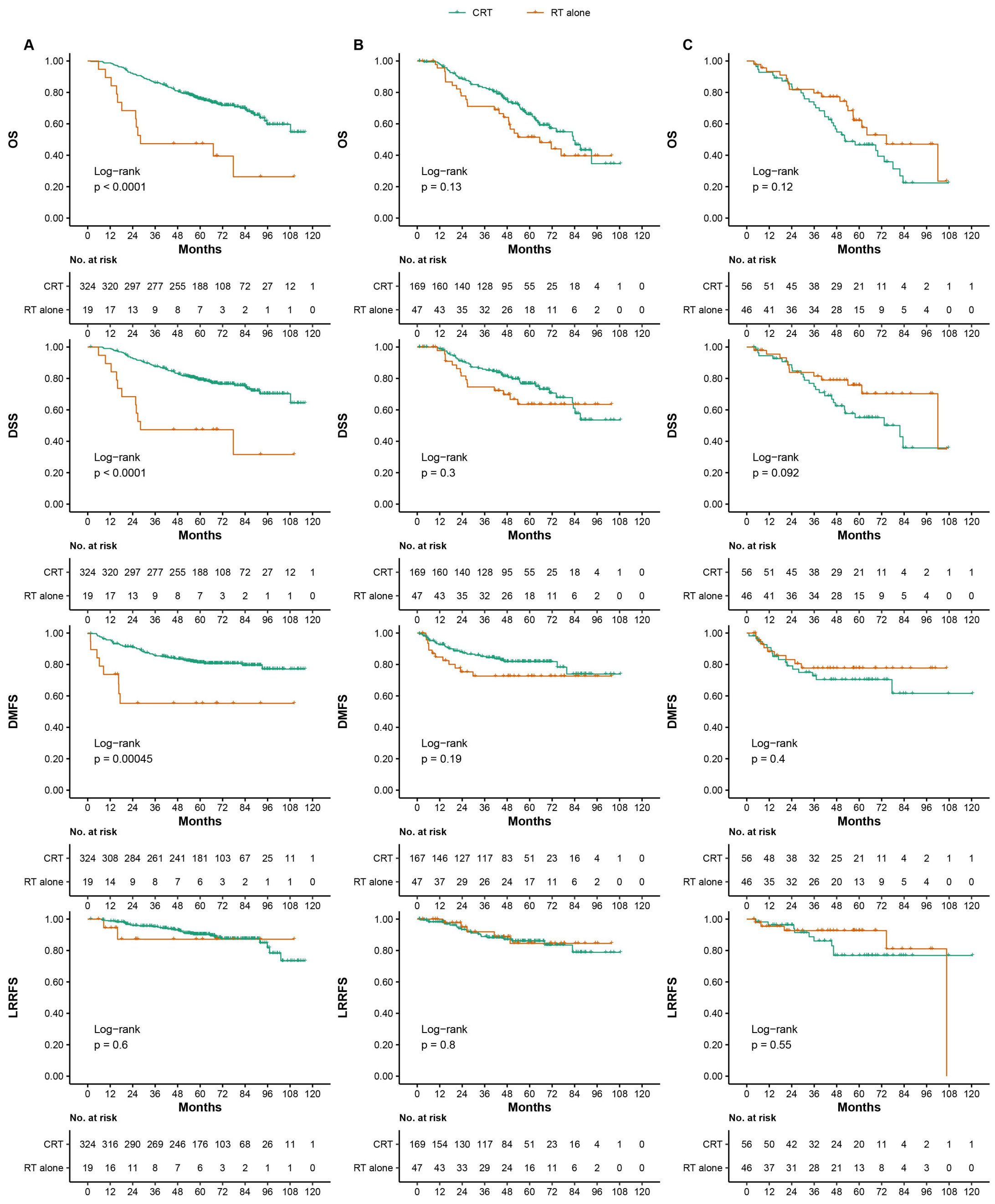

Supplementary Figure S8. Kaplan-Meier OS, DSS, DMFS and LRRFS curves in the high-risk group between CRT and RT alone for aged 60-64 years old (A), age 65-70 years old (B) and ≥70 years old (C). High-risk group: plasma EBV DNA titer ≥ 4,000 copies/mL & any T. EBV = Epstein-Barr virus; OS = overall survival; DSS = disease-specific survival; DMFS = distant metastasis-free survival; LRRFS = locoregional recurrence-free survival; CRT = chemoradiotherapy; RT = radiotherapy.

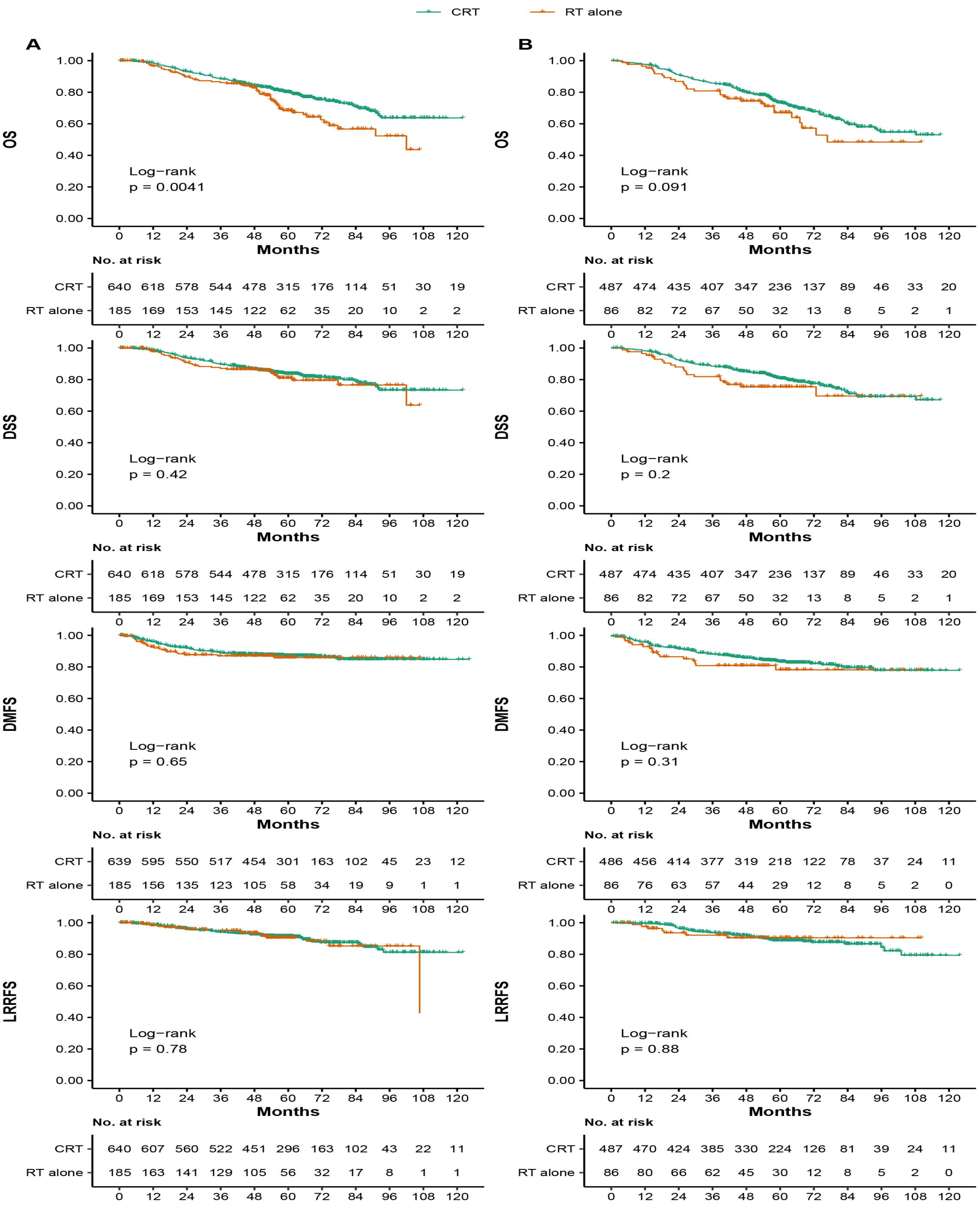

Supplementary Figure S9. Kaplan-Meier OS, DSS, DMFS and LRRFS curves in the poor-prognosis group between CRT and RT alone with non-smoking (A) and smoking (B). Poor-prognosis group = intermediate-risk group + high-risk group (intermediate-risk group: plasma EBV DNA titer ≤4,000 copies/mL & T3-4; high-risk group: plasma EBV DNA titer > 4,000 copies/mL & any T). EBV = Epstein-Barr virus; OS = overall survival; DSS = disease-specific survival; DMFS = distant metastasis-free survival; LRRFS = locoregional recurrence-free survival; CRT = chemoradiotherapy; RT = radiotherapy.

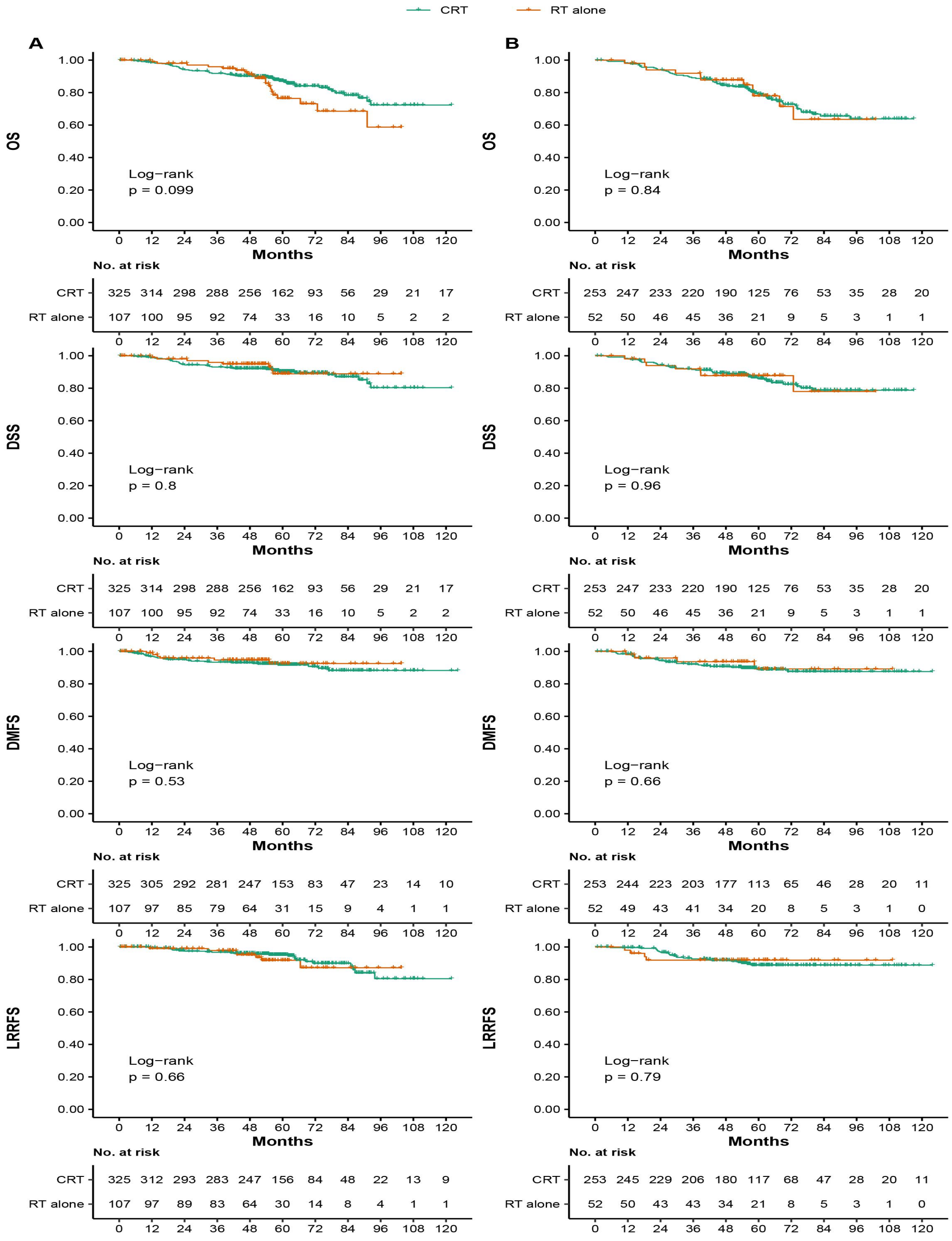

Supplementary Figure S10. Kaplan-Meier OS, DSS, DMFS and LRRFS curves for the intermediate-risk group between CRT and RT alone with non-smoking (A) and smoking (B). Intermediate-risk group: plasma EBV DNA titer ≤4,000 copies/mL & T3-4. EBV = Epstein-Barr virus; OS = overall survival; DSS = disease-specific survival; DMFS = distant metastasis-free survival; LRRFS = locoregional recurrence-free survival; CRT = chemoradiotherapy; RT = radiotherapy.

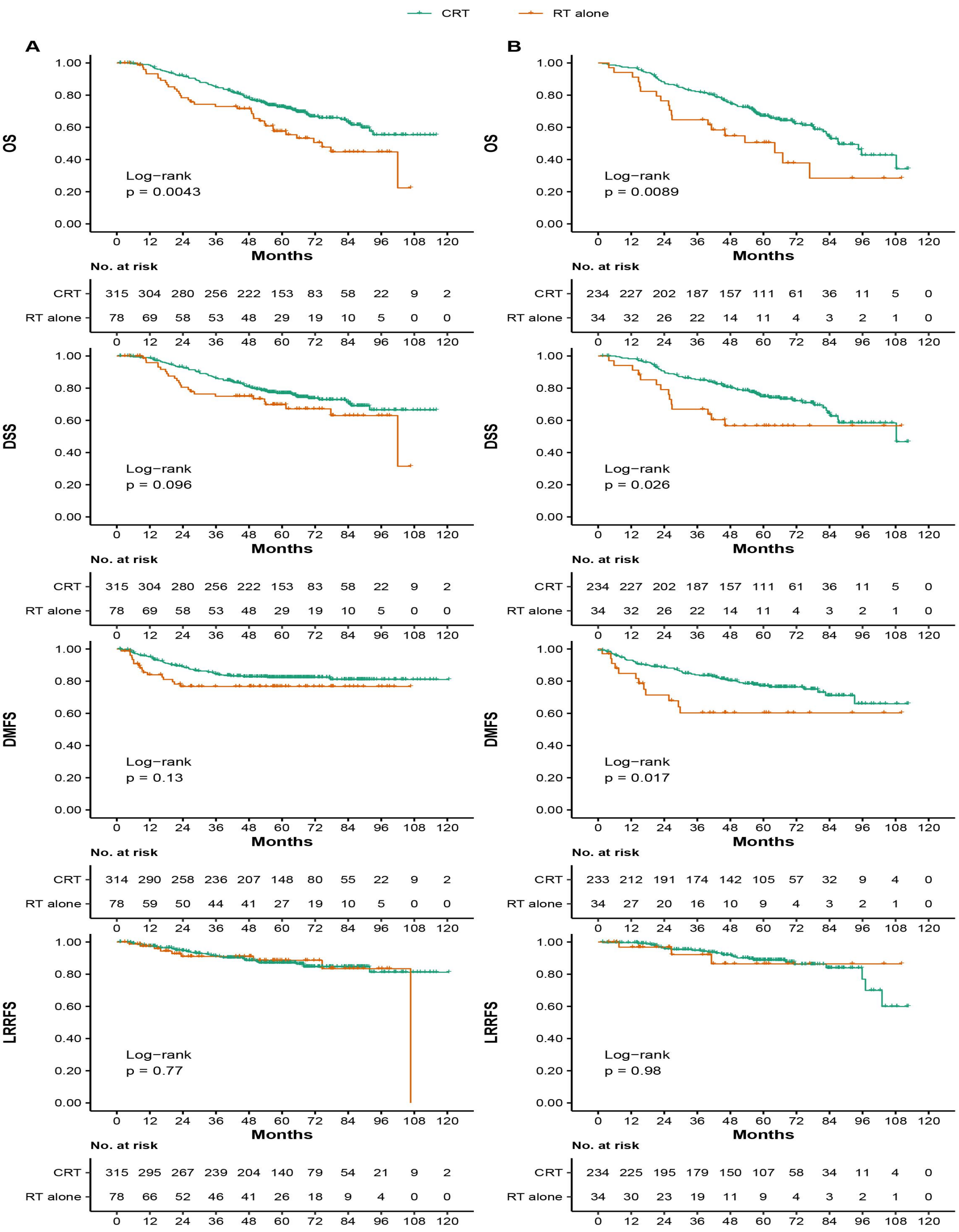

Supplementary Figure S11. Kaplan-Meier OS, DSS, DMFS and LRRFS curves for the high-risk group between CRT and RT alone with non-smoking (A) and smoking (B). High-risk group: plasma EBV DNA titer > 4,000 copies/mL & any T. EBV = Epstein-Barr virus; OS = overall survival; DSS = disease-specific survival; DMFS = distant metastasis-free survival; LRRFS = locoregional recurrence-free survival; CRT = chemoradiotherapy; RT = radiotherapy.

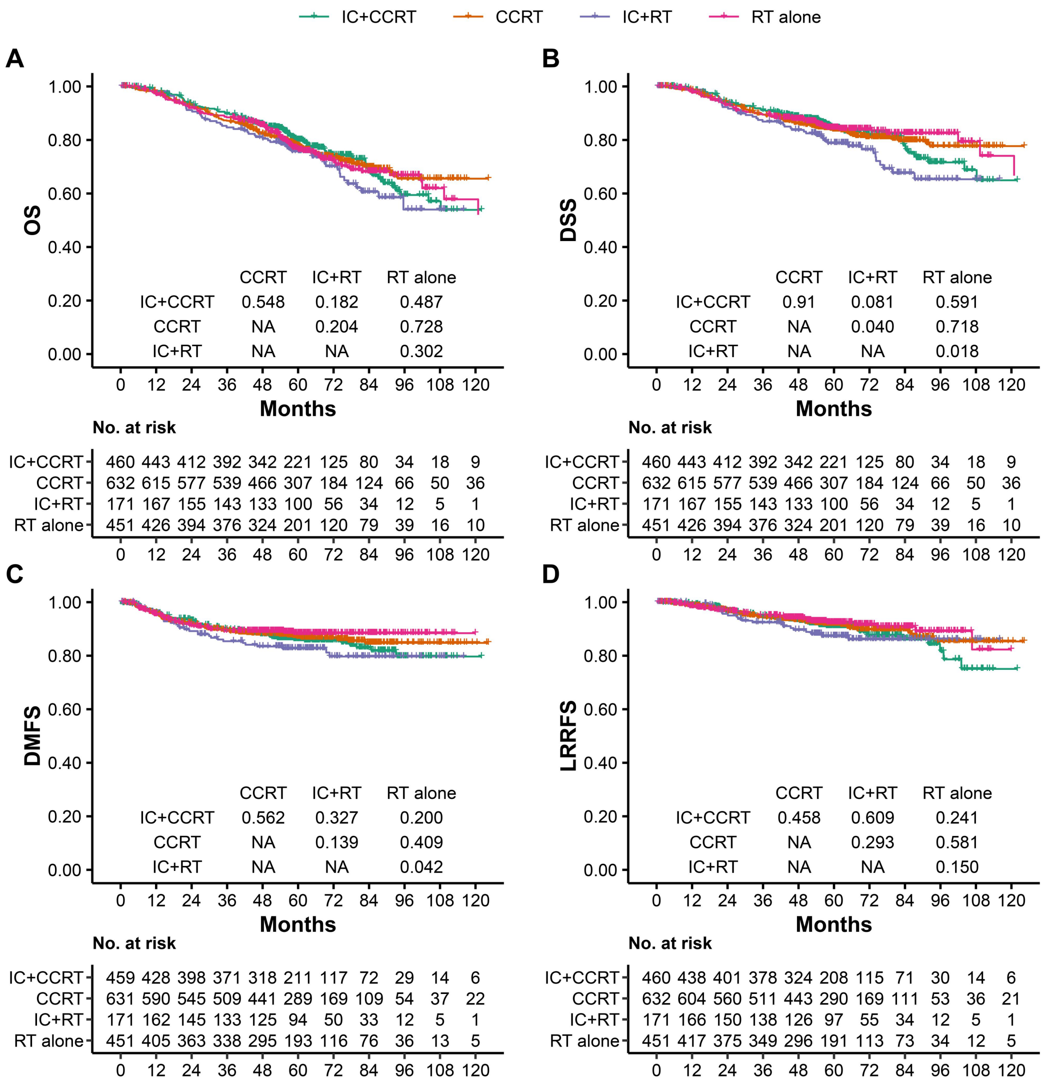

Supplementary Figure S12. Kaplan-Meier OS, DSS, DMFS and LRRFS curves between IC+CCRT, CCRT, IC+RT and RT alone for all of the elderly NPC patients. OS = overall survival; DSS = disease-specific survival; DMFS = distant metastasis-free survival; LRRFS = locoregional recurrence-free survival; CCRT = concurrent chemoradiotherapy; IC = induction chemotherapy; RT = radiotherapy.

SUPPLEMENTARY TABLES

Supplementary Table E1

Baseline characteristics for elderly NPC patients in the poor prognosis group.

	**CRT（n = 1,127）**	**RT（n = 271）**	***P***
**Characteristics**	**No. Patients (%)**	**No. Patients (%)**	
Gender			
male	876 (77.7)	199 (73.4)	0.154
female	251 (22.3)	72 (26.6)	
Age at diagnosis, years old			
60-64	648 (57.5)	58 (21.4)	<0.001
65-69	358 (31.8)	96 (35.4)	
≥70	121 (10.7)	117 (43.2)	
Histological type			
WHO type I–II	24 (2.1)	6 (2.2)	1
WHO type III	1103 (97.9)	265 (97.8)	
ACE-27, scores			
0	591 (52.4)	117 (43.2)	<0.001
1	482 (42.8)	101 (37.3)	
2	54 (4.8)	53 (19.6)	
Plasma EBV DNA titer, copies/mL			
≤ 4,000	578 (51.3)	159 (58.7)	0.034

＞4,000	549 (48.7)	112 (41.3)	
T category			
T1-2	97 (8.6)	26 (9.6)	0.692
T3-4	1,030 (91.4)	245 (90.4)	

Note: T category were determined based on the 8th Edition of American Joint Committee on Cancer/International Union Against Cancer staging system.

Abbreviations: plasma EBV DNA = plasma Epstein-Barr Virus DNA; WHO = World Health Organization; ACE-27 = Adult Comorbidity Evaluation 27; CRT = chemoradiotherapy; RT = radiotherapy.

*P values were calculated with univariate Cox proportional-hazards model.

Supplementary Table E2

Detailed induction and concurrent chemotherapy regimens for elderly NPC patients in the poor-prognosis group.

Characteristics	**Poor-prognosis group** CRT (n = 1,127) No. Patients (%)	**Intermediate-risk group** CRT (n = 578) No. Patients (%)	**High-risk group** CRT (n = 549) No. Patients (%)
IC regimens			
None	585 (51.9)	332 (57.4)	253 (46.1)
TPF	239 (21.2)	114 (19.7)	125 (22.7)
PF	119 (10.6)	53 (9.2)	66 (12.1)

TP	129 (11.4)	56 (9.7)	73 (13.3)
GP	55 (4.9)	23 (4)	32 (5.8)
CCRT regimens			
None	156 (13.8)	87 (15.1)	69 (12.5)
DDP	871 (77.3)	436 (75.4)	435 (79.3)
Nedaplatin	100 (8.9)	55 (9.5)	45 (8.2)

Abbreviations: poor-prognosis group = intermediate-risk group + high-risk group (intermediate-risk group: plasma EBV DNA titer ≤ 4,000 copies/mL & T3-4; high-risk group: plasma EBV DNA titer > 4,000 copies/mL & any T). NPC = nasopharyngeal carcinoma; IC = induction chemotherapy; CCRT = concurrent radiochemotherapy; CRT = chemoradiotherapy; DDP = cisplatin; TPF, cisplatin (60 mg/m^2) with 5-fluorouracil (600 mg/m^2 over 120 h), and docetaxel (60 mg/m^2); PF, cisplatin (80 mg/m^2) with 5-fluorouracil (800 mg/m^2/day over 120 h); TP, cisplatin (80 mg/m^2) with docetaxel (80 mg/m^2); GP, gemcitabine (1 g/m^2, d1,8) and cisplatin (80 mg/m^2).
